# *Salix tetrasperma* Roxb. Extract Alleviates Neuropathic Pain in Rats via Modulation of the NF-κB/TNF-α/NOX/iNOS Pathway

**DOI:** 10.3390/antiox8100482

**Published:** 2019-10-12

**Authors:** Mansour Sobeh, Mona F. Mahmoud, Samar Rezq, Amira E. Alsemeh, Omar M. Sabry, Islam Mostafa, Mohamed A. O. Abdelfattah, Khadija Ait El-Allem, Assem M. El-Shazly, Aziz Yasri, Michael Wink

**Affiliations:** 1 Institute of Pharmacy and Molecular Biotechnology, Heidelberg University, 69120 Heidelberg, Germany; 2 AgroBioSciences Research Division, Mohammed VI Polytechnic University, Lot 660–Hay MoulayRachid, Ben-Guerir 43150, Morocco; Khadija.AITELALLEM@um6p.ma (K.A.E.-A.); aziz.yasri@um6p.ma (A.Y.); 3 Department of Pharmacology and Toxicology, Faculty of Pharmacy, Zagazig University, Zagazig 44519, Egypt; mona_pharmacology@yahoo.com (M.F.M.); samar_rezq@yahoo.com (S.R.); 4 Department of Anatomy and Embryology, Faculty of Medicine, Zagazig University, Zagazig 44519, Egypt; dr_amira_2008@yahoo.com; 5 Department of Pharmacognosy, College of Pharmacy, Cairo University, Cairo 11562, Egypt; omar.sabry@pharma.cu.edu.eg; 6 Department of Pharmacognosy, Faculty of Pharmacy, Zagazig University, Zagazig 44519, Egypt; islam_mostafa_elbaz@yahoo.com (I.M.); assemels2002@yahoo.co.uk (A.M.E.-S.); 7 College of Engineering and Technology, American University of the Middle East, Egaila 54200, Kuwait; mohamed.abdelmoety@aum.edu.kw

**Keywords:** *Salix tetrasperma*, polyphenols, neuropathy, NF-κB/TNF-α/NOX/iNOS pathway, chronic constriction injury, p53

## Abstract

Patients with neuropathic pain experience chronic painful tingling, burning, and prickling sensations accompanied with hyperalgesia and/or allodynia. In this study, 38 secondary metabolites of a methanol extract from *Salix tetrasperma* flowers were identified by liquid chromatography-mass spectrometry (HPLC-MS/MS). The extract showed substantial anti-inflammatory, central and peripheral anti-nociceptive, antipyretic, and antioxidant activities in vitro and in different animal models. In the chronic constriction injury (CCI) rat model, the extract was able to attenuate and significantly relieve hyperalgesia and allodynia responses in a dose dependent manner and restore the myelin sheath integrity and Schwann cells average number in the sciatic nerve. The enzyme-linked immunosorbent assay (ELISA) showed that the extract significantly reduced the expression of various pro-inflammatory biomarkers including nuclear factor kabba B (NF-κB), tumor necrosis factor alpha (TNF-α), prostaglandin E2 (PGE2), 5-lipoxygenase (5-LOX), cyclooxygenase-2 (COX-2), inducible nitric oxide synthase (iNOS), and the oxidative stress biomarker NADPH oxidase 1 (NOX1), in brain stem and sciatic nerve tissues. These findings were supported by in vitro enzyme inhibition assays (COX-1, COX-2 and 5-LOX). Moreover, the extract significantly reduced p53 expression in the brain stem tissue. These findings support the use of *S. tetrasperma* in folk medicine to alleviate pain. It could be a promising natural product for further clinical investigations to treat inflammation, nociceptive pain and chronic neuropathic pain.

## 1. Introduction

Neuropathic pain is defined as unpleasant sensations of burning and tingling with increased sensitivity towards pain. This pain arises as a direct response to a lesion or disease affecting the somatosensory system. As a result, neuropathic pain and pathological conditions may appear spontaneously or as amplified responses to noxious and innocuous stimuli [1].

Various ailments are associated with neuropathic pain such as infections, malignancy, trauma, toxicity of certain medications and toxins, and some metabolic diseases. Patients with diabetes, AIDS, stroke, and spinal cord injury may develop peripheral neuropathic pain as well [2]. Neuropathic pain is mostly accompanied by two classic symptoms, hyperalgesia (increased sensitivity to painful stimuli) and allodynia (pain due to a stimulus which does not normally provoke pain) [1].

Several models are used to evaluate the treatments used to counter neuropathic pain. The most common one is the chronic constriction induced sciatic nerve injury (CCI) in rats that creates a cutaneous sensory threshold following the injury and displays allodynia to noxious mechanical stimuli and hyperalgesia to harmful heat stimuli. The model was reported to exhibit similar etiologies to those detected in several clinical conditions including stroke, pain syndrome, and arthroplasty [2].

Antidepressants and anticonvulsants are commonly used for treating neuropathic pain. However, they show only partial relief and are accompanied by many adverse effects [3]. Medicinal plants have been used in folk medicine to counter pain and thus could represent a promising alternative for alleviating neuropathic pain [4,5].

The Indian willow, *Salix tetrasperma* Roxb., (family Salicaceae) is native to South East Asia, and India. Its bark has been used in traditional medicine in many countries to alleviate pain, fever, and inflammation. Previous studies have identified many phytochemicals in the bark extract such as flavonoids, tannins, and saponins. The anti-inflammatory activity is mainly attributed to salicin, which is a prodrug for salicylic acid that inhibits the cyclooxygenase, a key enzyme in the inflammation pathway [5,6]. The analgesic activity of *S. tetrasperma* bark extract was evaluated by acetic acid induced writhing method and showed that the extract clearly possessed a pain-relieving effect [6].

The present study characterized the active principles present in a flower extract using by liquid chromatography-mass spectrometry (HPLC-MS/MS). The antipyretic, anti-inflammatory and analgesic activities of the extract in addition to its effect on the development of hyperalgesia and allodynia behavior in the rat CCI model of neuropathic pain were studied. The effect of the extract on the expression of inducible nitric oxide synthase (iNOS), prostaglandin E2 (PGE2), NADPH oxidase 1 (NOX1), cyclooxygenase-2 (COX-2), 5-lipoxygenase (5-LOX), catalase, tumor necrosis factor alpha (TNF-α), NF-κB, and p53 was also investigated. Enzyme inhibitory assays for COX-1, COX-2 and 5-LOX, in vitro antioxidant assays were performed to further confirm the obtained results. 

## 2. Materials and Methods

### 2.1. Plant Material and Extraction

The flowers (catkins) of *S. tetrasperma* Roxb. were collected during the spring season (30 April 2018) from the province of Qalubiya, (Banha-Zagazig road, location 30°28′15″ N 31°14′50″ E), Egypt. Plant identity was verified morphologically, and a voucher specimen was deposited in the Herbarium of Pharmacognosy Department, Faculty of Pharmacy, Zagazig University, Egypt (Voucher specimen No. SST-3, Figure S1). The shade dried flowers (200 g) were ground into fine powder by electric mill and extracted twice with methanol (2 × 1 L) with occasional shaking at room temperature for three days. The combined extracts were filtered and concentrated at 40 °C to yield 44 g of the crude extract (yield: 22% *w/w*). The obtained extract was defatted with hexane, frozen, and lyophilized yielding a fine powder (38 g) then stored at −20 °C for chemical and biological investigations.

### 2.2. In Vitro Experiments

In vitro antioxidant activities, cyclooxygenase (COX) and lipoxygenase (LOX) inhibition assays were carried out as previously described [7]. Detailed methods are included in the supplementary file.

### 2.3. In Vivo Experiments

#### 2.3.1. Animals

Adult male Wistar rats and Swiss albino mice (Faculty of Veterinary Medicine, Zagazig University, Zagazig, Egypt) weighing 140–160 g and 20–25 g, respectively were housed under a controlled temperature (22 °C) and humidity (50 ± 10%) at 12 h light/dark cycle with food (regular chow) and water supplied ad libitum. Experimental design and treatment protocols were approved by the Ethical Committee for Animal Handling at Zagazig University (ECAH ZU), Faculty of Pharmacy, Zagazig University, Egypt (approval number: ZU-IACUC/3/F/115/2018) and in compliance with the recommendations of the Weatherall report. 

Evaluating the in vivo anti-inflammatory, anti-nociceptive and antipyretic activities was carried out as previously described [7]. The carrageenan-induced hind-paw edema in rats, recruitment of leukocyte to peritoneal cavity and acetic acid-induced vascular permeability in mice models were used to investigate the anti-inflammatory activity of the extract at different dose levels (200, 400 and 600 mg/kg, p.o.). The acetic acid induced writhing and hot plate test models in mice were used to investigate the extract’s anti-nociceptive activity. To study the antipyretic effect, the Brewer’s yeast model of pyrexia in mice was used. Detailed methods are included in the supplementary file.

#### 2.3.2. Induction of Neuropathic Pain by Chronic Constriction Injury (CCI)

Sciatic nerve CCI was induced unilaterally in rats as previously reported [8]. Following anesthesia with thiopental (50 mg/kg, ip), the right sciatic nerve was exposed under aseptic conditions. The CCI was induced by placing four loose and 1 mm apart ligatures (4/0 silk suture) around the nerve. The wound was then closed, and the animals were placed into their cages to recover. In the sham group, the right sciatic nerve was exposed without constriction. The behavioral tests were performed on day 1 before the surgery and at days 7 and 14 after surgery. The animals received the different treatments daily after the surgery for the whole experiment duration. At day 14 and after the last behavioral test was performed, the animals were euthanized then blood and tissues were collected for the different biochemical measurements as described below.

#### 2.3.3. Behavioral Examination

Behavioral tests were conducted by a blinded experimenter.

#### 2.3.4. Mechanical Hyperalgesia (Pinprick Test)

Mechanical hyperalgesia was assessed using the pinprick test as previously described by [9]. The tip of bent needle gauge was used to touch the injured hind paw avoiding skin penetration that led to a reflex withdrawal response. The duration of paw withdrawal was recorded in seconds. The cut-off time of the test was set at 20 s. and a value of 0.5 s was given if a brief normal response was observed. 

#### 2.3.5. Mechanical Dynamic Allodynia (Paint-Brush Test)

The term allodynia can be defined as painful response to stimulus that is normally painless such as smooth paint brush. In this model a paint brush was used to test the dynamic responses of the injured paw to a mechanical stimulus. The rat was placed on an elevated wire mesh floor, covered with a glass cage. The brush was then used to rub the hind paw plantar area from the heel to the toes five times separated by a 5 s interval. The number of withdrawals (between 0 and 5) was observed. The test was repeated twice, with a 5 min rest period. The mechanical dynamic allodynia score will be the sum of total number of withdrawals obtained of the 15 trials (between 0 and 15) [10,11]. 

#### 2.3.6. Heat Hyperalgesia (Hot Plate Test)

Hot plate test was used to assess the possible analgesic effect of the extract against thermal hyperalgesia as previously reported [12]. The rat was placed on the hot plate at a temperature of 52.5 ± 1.0 °C and the time to the first sign of hyperalgesia (shaking, paw withdrawal, paw licking, or jumping) was recorded in seconds with a cut-off time of 20 s to avoid potential tissue damage.

#### 2.3.7. Paw Cold-Allodynia (Acetone Drop Test)

The ability of the extract to protect rats against cold allodynia in CCI model was assessed by exposing the injured paw to a cold stimulus (spraying 100 μL of acetone) and observing the response (graded on a 4-point scale) for 20 s. The responses were recorded as per the following: 0, no response; 1, quick withdrawal, flick or stamping the paw; 2, prolonged withdrawal or repeated flicking; and 3, repeated flicking with paw licking. The procedure was repeated three times at 5 min intervals. The final score (from 0 to 9) equals to the summed-up scores of the three trials [13].

#### 2.3.8. Histopathological Examination

Histopathological examination was performed according to [14]. Brains and sciatic nerves (*n* = 5 each) were fixed in 10% neutral buffered formalin and then embedded in paraffin. Paraffin sections (6 sections/animal, 5 μm thickness) were deparaffinized in xylene before staining with hematoxylin and eosin (H&E). Images were analyzed by light microscopy (LEICA ICC50 W). The Nerve Injury Scoring System (NISS) was used to assess the degree of Myelin damage according to the following: 1, normal, mild degeneration or demyelination; 2, moderate level of degeneration (<50% damaged) and 3, diffused degeneration or demyelination with >50% damaged nerve tissue [15].

#### 2.3.9. Immunohistochemical Staining of p53

A streptavidin-biotin complex immunoperoxidase system was used to detect p53 antibodies. The specimen sections were deparaffinized, incubated for 30 min in 0.1% hydrogen peroxide to block the endogenous peroxidase then rinsed three times with phosphate-buffered saline (PBS). Antigen retrieval was done using microwave treatment (20 min, 0.01 mol/L citrate buffer, pH 6). The slides were the incubated overnight with rabbit monoclonal p53 Antibody (monoclonal mouse anti-human p53 protein; clone DO-7; N1581; 10041283; 11 mL, 1:200), at 4 °C. The sections were then washed with PBS and incubated for 30 min with biotinylated anti-rabbit antibody (Versal kits, Zymed laboratories, Inc., South San Francisco, CA, USA, 1:200) at room temperature. Mayer’s hematoxylin dye was used to counterstain the sections which were then dehydrated and mounted. Another section was incubated with PBS instead of the primary antibody and served as negative control. Brown staining of the nuclei was considered as positive reaction for p53 [16]. The number of p53 positive cells was counted by Image J analysis software (Fiji image j; 1.51 n, NIH, Bethesda, MA, USA) in 3 non overlapping perceptive fields at 200% magnification from 6 sections/animal (5 animals/group). The data was expressed as the mean values of the p53 immunopositive nuclei percentage to the total nuclei in the field.

#### 2.3.10. Behavioral and Neurological Toxicity Assessment

##### Open-Field Activity

The effect of the extract (200, 400 and 800 mg/kg, p.o.) on locomotor activity (number of lines crossed in 3 min), anxiety like (time spent in center in seconds) and exploratory behaviors were assessed using the open-field test as previously described [17,18]. The animal was placed in the center of an open field wooden box (40 cm × 40 cm × 40 cm) divided into sixteen equal parts (10 × 10 cm each) with black lines and allowed to freely move for 3 min. The time spent in center and the number of lines crossed during the recording period was detected.

##### Emotionality 

The number of feces and urination as a measure for emotionality was recorded during the 3 min observation period in the open field test as previously reported [19].

##### Elevated Plus Maze

This test was performed to assess anxiety-like behavior in mice treated with the extract (200, 400 and 800 mg/kg, p.o.) for 14 days. Briefly, the animal was gently put into the center of the apparatus with its head facing the open arm. Each animal was observed for 5 min and the number of entries and the time spent in each arm was recorded and expressed as % of total time [18].

##### Rotarod Test

Rotarod test was utilized to evaluate the effect of 14 days administration of the extract (200, 400 and 800 mg/kg, p.o.) on motor coordination and balance. The mice were put on a rotating rotarod that was set to accelerate from 0 to 40 rpm in 120 s and the time in seconds before the mouse falls was recorded in each trial (three total). A time of 300 s was set as the time limit of each trial [20]. 

#### 2.3.11. Chronic Ulcerogenic Activity

The ulcerogenic potential of the extract was investigated and compared to indomethacin and celecoxib as reference drugs. At the end of the treatment period, the animals were euthanized, and stomachs were collected, washed with normal saline (0.9%) solution and examined for ulceration. The ulcer severity scores were calculated according to [21] and [22] as follows: 0, normal colored stomach; 0.5, red coloration; 1, spot ulcer; 1.5, hemorrhagic streaks; 2, an ulcer > 3 but <5 mm in diameter; and 3, an ulcer > 5 mm in diameter. The ulcer index (UI) was calculated using the equation:

[UI = UN + US + UP × 10^−1^], (1)

where UN is the average ulcers number, US is the average severity score, and UP is the percentage of animals with an ulcer.

### 2.4. Biochemical Measurements

The involvement of both peripheral and central components in chronic neuropathic pain progression necessitates investigating the anti-inflammatory potential of the extract both in sciatic nerve and in the brain stem, which is known to be greatly involved [23]. Brain stems and sciatic nerve tissues obtained from different animals were homogenized in PBS followed by centrifugation at 14,000 rpm for 20 min at 4 °C. The protein content of the supernatant was determined using Bradford assay (Bio-Rad, Hercules, CA, USA). Inducible nitric oxide synthase (iNOS) and prostaglandin E2 (PGE2) were measured using rat ELISA Kits obtained from mybiosource (San Diego, CA, USA) and Cayman (Ann Arbor, MI, USA), respectively. NADPH oxidase 1 (NOX1), cyclooxygenase-2 (COX-2), 5-lipoxygenase (5-LOX), catalase, TNF-α, and NF-κB were detected using ELISA Kit obtained from Cusabio (Houston, TX, USA) according the manufactures’ instruction.

### 2.5. Statistical Analysis

Data are expressed as the mean ± SEM. Multiple comparisons were performed using GraphPad Prism Version 6.01 (GraphPad Software, San Diego, CA, USA) by one-way analysis of variance (ANOVA) or repeated-measures analysis of variance (RM-ANOVA). Post hoc analysis (Tukey multiple comparison test) or Student’s t-test was used to detect difference among groups. A *p* value < 0.05 was considered statistically significant.

## 3. Results

HPLC-MS/MS analysis documented the presence of 38 secondary metabolites in the methanol extract of *S. tetrasperma* flowers. Rutin, kaempferide 3-*O*-glucoside, trichocarposide, coumaroylquinic acid and salicin dominated the extract (Table 1, Figure 1 and Figures S2–S18).

### 3.1. In Vitro Effects of the Extract on COX-1, COX-2, 5-LOX and Total Antioxidant Capacity

As shown in Table 2, the extract inhibited both COX-1 and COX-2 in vitro with a higher selectivity towards COX-2. The total antioxidant capacity (TAC) of the extract was comparable to that of ascorbic acid, the reference antioxidant standard, Table 2.

### 3.2. Effects on Carrageenan-Induced Paw Edema in Rats

Rats injected with 0.1 mL carrageenan (1% in 0.9% NaCl, sub-planter) showed an increased paw thickness that was measured hourly for 5 h and at 24 h after injection. The maximum effect was observed 2 h post injection (3.5 ± 0.17 mm over initial paw thickness reading). On the other hand, rats given the extract (200 mg/kg, p.o.) 1 h earlier showed attenuated increase of edema development (evidenced as a decrease in the AUC_0–24_ values by nearly 35% of control values). Increasing the dose of the extract up to 600 mg/kg did not provide an advantage over the lowest used dose (Figure S19), whereas, the rats treated with the standard non-steroidal anti-inflammatory drug, diclofenac (20 mg/kg, p.o.) and the steroidal anti-inflammatory drug, dexamethasone (2 mg/kg, p.o.) had 52 and 45% edema reduction, respectively compared to control rats (Figure S19).

### 3.3. Effects of the Extract on Carrageenan-Induced Leukocyte Migration into the Peritoneal Cavity in Mice

As shown in Figure S20, mice injected with 0.1 mL carrageenan (500 μg/mouse, i.p.) showed an obvious leukocyte migration response into their peritoneal cavity. An increase in the total leukocyte number was observed compared to the saline treated mice (6.32 ± 1.12 vs. 1.03 ± 0.09 leukocytes × 10^6^ mL^−1^). Treatment with the extract (200, 400 and 600 mg/kg, p.o.) attenuated the carrageenan effect by 27%, 48% and 75%, respectively in a dose dependent manner. The effect of 400 mg/kg, p.o. dose was comparable to that of diclofenac (10 mg/kg, p.o.) which reduced the number of leukocytes by 49% (Figure S20).

### 3.4. Effects of the Extract on Acetic Acid-Induced Vascular Permeability in Mice

Mice injected with acetic acid (0.6%, i.p.) showed an increased vascular permeability represented as a significantly (*p* < 0.001) higher Evans blue absorbance (0.58 ± 0.11) of the peritoneal cavity exudate compared to the saline injected mice (0.07 ± 0.002). This response was significantly reduced upon 1 h prior treatment with the extract (200, 400 and 600 mg/kg, p.o.). All treatment doses resulted in nearly 67% reduction of control readings. The reference diclofenac achieved 62% lower reading compared to the control mice (Figure S21).

### 3.5. Effects of the Extract on Acetic Acid-Induced Writhing in Mice

As shown in Figure S22, oral pretreatment with the extract showed a remarkable analgesic activity in both doses (200 and 400 mg/kg, p.o.) represented as a decrease in acetic acid (0.7% acetic acid, 1 mL/100 g) induced writhes in mice by 77% and 83%, respectively. Mice pretreated with diclofenac (20 mg/kg, p.o.) and dexamethasone (2 mg/kg) showed a 64% and 72% reduction in writhes, respectively compared to the control (Figure S22a).

### 3.6. Effects of the Extract on Hot Plate Test in Mice

The response latency (measured at 2, 3 and 4 h after administration) to heat hyperalgesic stimulus was longer in animals pretreated with the extract (200 mg/kg, p.o.) compared to vehicle treated animals. The later peaked at the 3 h time point (3.1-fold of control). Noteworthy, using a higher dose of the extract (400 mg/kg) resulted in faster (occurred 1 h post-treatment) but relatively shorter effect (Figure S22b). 

### 3.7. Effect of the Extract on Brewer’s Yeast Induced Pyrexia in Mice

Mice injected with Brewer’s showed a higher rectal body temperature that was measured 18 h post yeast injection (Table 3). The extract (200 mg/kg, p.o.) showed a fast but brief antipyretic effect that occurred 30 min post-treatment. Increasing the dose of the extract to 400 mg/kg did not increase the duration of the antipyretic effect (Table 3). The reference paracetamol (150 mg/kg) showed a delayed but prolonged action (Table 3).

### 3.8. Effect of the Extract on Pain and Inflammation in CCI Model

#### 3.8.1. Effect on Heat Hyperalgesia and Cold Allodynia

**Figure 2:**
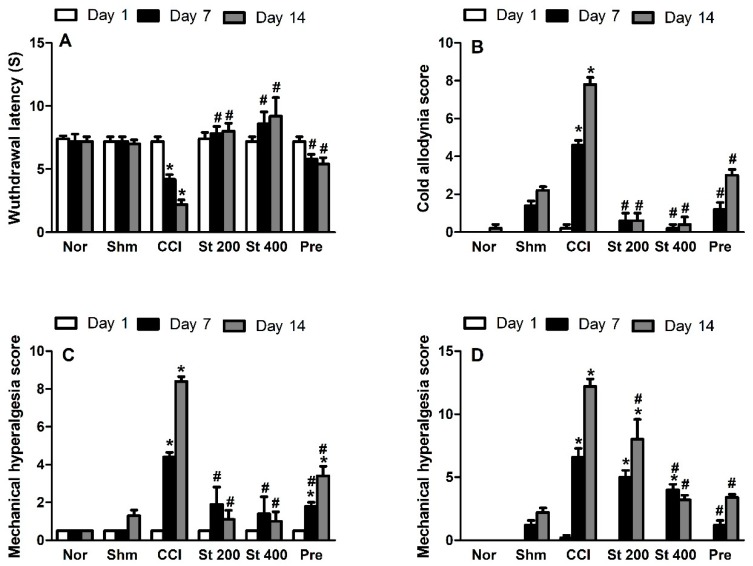
Effect of *S. tetrasperma* extract (200 and 400 mg/kg, p.o.) on (**A**) heat hyperalgesia (withdrawal latency) or (**B**) cold allodynia responses in rats with neuropathic pain induced by spinal nerve ligation (**C**) Effect of *S. tetrasperma* extract (200 and 400 mg/kg, p.o.) on mechanical hyperalgesia score as assessed by pin brick test in neuropathic pain rats induced by spinal nerve ligation. Paw withdrawal duration was recorded in seconds and ranges from 0.5 s for the brief normal response to 20 s (the cut-off time). (**D**) Effect of *S. tetrasperma* extract (200 and 400 mg/kg, p.o.) on mechanical dynamic allodynia as assessed by paint brush test in neuropathic pain rats induced by spinal nerve ligation. The number of withdrawals of total of 15 trials in response to a stimulus induced by paint brush was noted (between 0 and 15). Data is expressed as mean ± SE. * *p* < 0.05, compared to Sham group; # *p* < 0.05, compared to chronic constriction induced sciatic nerve injury (CCI) group at the corresponding time points. Norm means normal group; Shm means Sham group; St 200 and St 400 mean *S. tetrasperma* extract (200 and 400 mg/kg) and pre means pregabalin group.

Rats exposed to CCI showed signs of both heat hyperalgesia (Figure 2A) and cold allodynia (Figure 2B) responses represented as a dose dependent decrease and increase in heat response latency time and cold allodynia scores, respectively when compared to sham group. On the other hand, rats treated with the extract (200 and 400 mg/kg, p.o.) showed significantly lower heat hyperalgesia and cold allodynia responses when measured at days 7 and 14 post CCI. Noteworthy, the extract exhibited a stronger response than the standard pregabalin.

#### 3.8.2. Effect on Mechanical Hyperalgesia

CCI resulted in a significant (*p* < 0.001) increase in mechanical hyperalgesia assessed by pin brick test performed on days 7 and 14 post-surgery represented by an increase in the withdrawal time of the injured hind paw compared to the sham group by 8.8 and 7.6-fold, respectively (Figure 2C). This effect was attenuated in rats that received the extract (200 and 400 mg/kg p.o.), starting at day 7 post CCI. It was noted that the extract exhibited a stronger response than pregabalin. 

#### 3.8.3. Effect on Mechanical Dynamic Allodynia

As shown in Figure 2D, CCI resulted in a significant (*p* < 0.001) increase in dynamic allodynia scores assessed by paint brush test when measured at day 7 and 14 post-surgery compared to sham group (6.4 ± 0.75 vs. 1.2 ± 0.37 and 12.5 ± 0.51 vs. 2 ± 0.32), respectively. Rats treated with the extract (200 and 400 mg/kg, p.o.) showed attenuated dynamic allodynia response with the higher dose demonstrating a comparable effect to pregabalin.

#### 3.8.4. Effect on CCI-Induced Increase in COX-2, 5-LOX and PGE2

**Figure 3:**
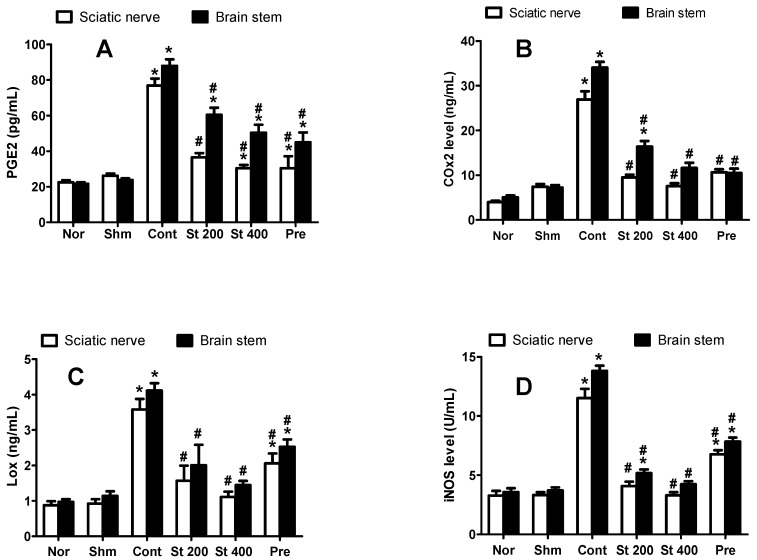
Effect of *S. tetrasperma* extract (200 and 400 mg/kg, p.o.) on CCI-induced rise in PGE2 (**A**), COX-2 (**B**) and 5-LOX (**C**) and iNOS levels in the sciatic nerves and brain stems of CCI rats (**D**). Values are given as mean ± SE, *n* = 5 rats per group. * *p* < 0.05, compared to Sham group; # *p* < 0.05, compared to CCI group. PGE2, prostaglandin E2; COX-2, cyclooxygenase 2; 5-LOX, lipoxygenase, iNOS, inducible nitric oxide synthase. Norm means normal group; Shm means Sham group; St 200 and St 400 mean *S. tetrasperma* extract (200 and 400 mg/kg) and pre means pregabalin group.

CCI (measured on day 14) significantly (*p* < 0.05) increased sciatic nerve and brain stem COX-2 (3.6 and 4.7-fold), 5-LOX (3.4 and 4.2-fold) and PGE2 (3 and 3.7-fold) levels, respectively, compared to that of sham rats. On the other hands, sciatic nerves and brain stems obtained from rats treated with the extract (200 and 400 mg/kg, p.o.) for 14 days showed significantly lower levels of COX-2 with 35% and 28%, respectively of the control sciatic nerves and 48% and 34%, respectively of the control brain stems. Furthermore, 5-LOX levels were significantly reduced by 56% and 69% of control values in sciatic nerve and by 51% and 65% in brain stem, respectively. Finally, the extract (200 and 400 mg/kg, p.o.) reduced PGE2 levels in the sciatic nerve by 53% and 60%, respectively, and in the brain stem by 32% and 43%, respectively compared to the control group (Figure 3A–C).

#### 3.8.5. Effect on CCI-Induced Increase in iNOS

As shown in Figure 3D, CCI resulted in significantly elevated levels of brain stem (13.82 ± 0.44 vs. 3.7 ± 0.2) and sciatic nerve (11.5 ± 0.78 vs. 3.3 ± 0.22) iNOS compared to sham group. This was attenuated when CCI rats were treated with the extract (200 and 400 mg/kg, p.o.) for 14 days by 63% to 71% compared to the vehicle treated rats.

#### 3.8.6. Effect on CCI-Induced Oxidative Status

**Figure 4:**
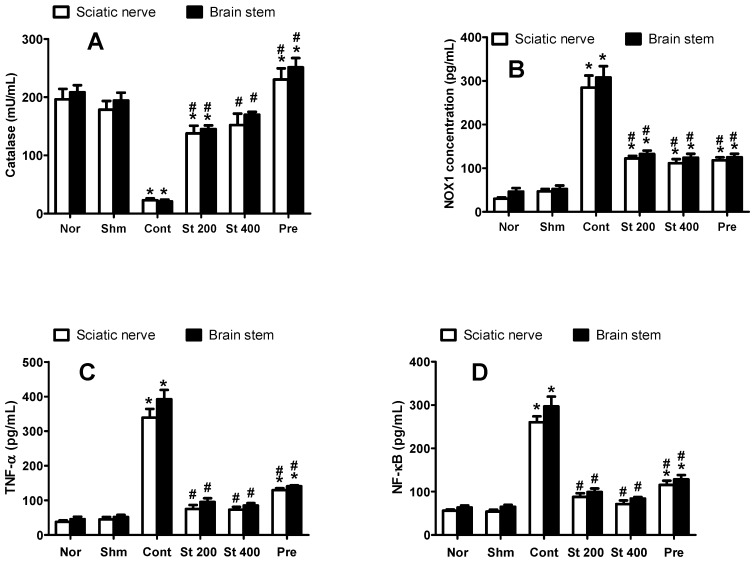
Effect of *S.* *tetrasperma* extract (200 and 400 mg/kg, p.o.) on CCI-induced on catalase activity (**A**), NOX1 level (**B**), TNF-α (**C**) and NF-κB (**D**) levels in the sciatic nerves and brain stems of CCI rats. Values are given as mean ± SEM, *n* = 5 rats per group. * *p* < 0.05, compared to Sham group; # *p* < 0.05, compared to CCI group. NOX1, NADPH oxidase; NF-κB, nuclear factor kappa B; TNF- α, tumor necrosis factor alpha. Norm means normal group; Shm means Sham group; St 200 and St 400 mean *S. tetrasperma* extract (200 and 400 mg/kg) and pre means pregabalin group.

CCI resulted in an increased oxidative status indicated by significantly (*p* ˂ 0.0001) higher levels of NADPH oxidase (NOX1) and lower levels of the antioxidant enzyme catalase compared to sham group when measured in brain stems and sciatic nerves of CCI rats. As shown in Figure 4, CCI rats treated with the extract (200 and 400 mg/kg, p.o.) showed an improved oxidative status represented as decreased NOX1 activity and an increase in catalase activity measured in both preparations compared to sham rats.

#### 3.8.7. Effect on NF-κB and TNF-α

Rats exposed to CCI for 14 days showed significantly elevated levels of sciatic and brain stem NF-κB (4.8 and 4.6-fold, respectively) and TNF-α (7.5-fold) compared to sham group. Rats treated with the extract (200 and 400 mg/kg, p.o, 14 days) significantly attenuated the CCI-induced increase in NF-κB by 66–72%. Similar results were obtained in TNF-α levels where lower levels were observed in the brain stems and sciatic nerves of the extract (200 and 400 mg/kg) treated rats (by 76–78%) compared to control group, Figure 4.

### 3.9. Effect on Expression of the Apoptotic Protein p53

**Figure 5:**
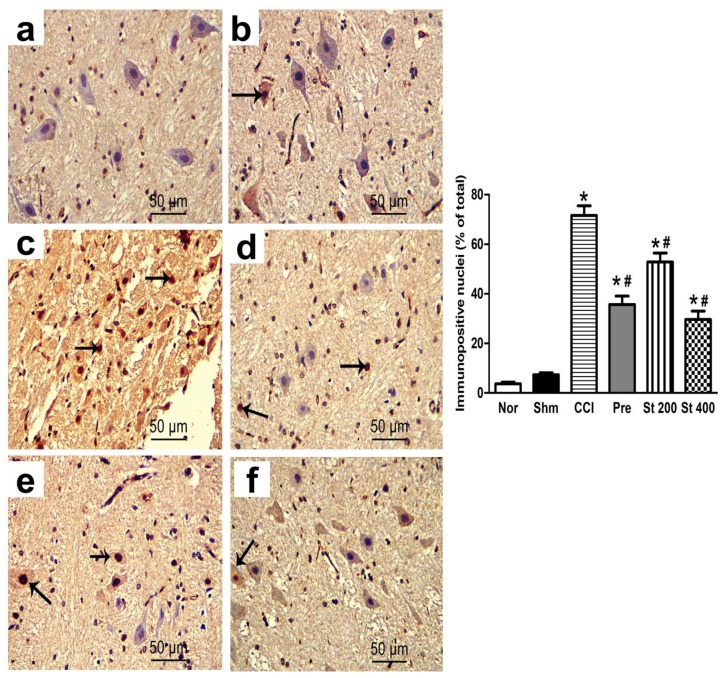
Representative photomicrographs showing the evaluation of the apoptotic cell populations via P53 (**a–f**) immunostaining of brain stems in: Normal (**a**); Sham (**b**); CCI (**c**); Pregabalin (**d**) *S. tetrasperma* (200 mg/kg) (**e**); *S. tetrasperma* (400 mg/kg) (**f**). Rats received different treatments for 14 days after which the animals were sacrificed, and tissues were collected. Arrows indicating dark brown staining of immunopositive cells nuclei. Scale bar; 50 μm. Bar chart showing changes in the number of p53 positive cells (% of total) in the brainstem sections of all experimental groups. The immunopositive cells were counted in 3 non overlapping fields from 100% magnification (*n* = 5 animals/group). Scale bar, 50 μm. * *p* < 0.05 vs. control values; # *p* < 0.05 vs. CCI group. Norm means normal group; Shm *means* Sham group; St 200 and St 400 mean *S. tetrasperma* extract (200 and 400 mg/kg) and pre means pregabalin group.

Brain stem tissues were immunohistochemically stained with anti-p53 antibody to assess the apoptotic neuron populations in all investigated groups. The p53-positive neurons were identified as those with dark brown nuclei. They were negative in the control group and infrequently noticed in the sham group, which revealed no significant difference with the control group. In contrary, the immunopositive cells were obviously detected in the CCI group which showed statistically significant increment compared to control group. Brain stems of rats treated with the extract (200 and 400 mg/kg, p.o.) showed significantly lower number of positive cells in a dose dependent manner compared to the control group. This effect was comparable to the pregabalin group, as seen in Figure 5.

### 3.10. Histopathological Results

#### 3.10.1. Effect on Sciatic Nerves

**Figure 6:**
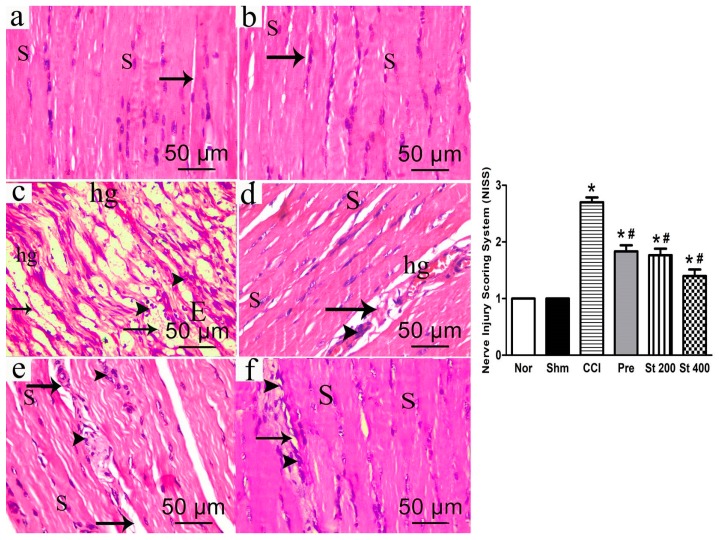
Upper panels: Photomicrograph (hematoxylin and eosin (H&E × 400) of longitudinal sciatic nerve sections from: Normal (**a**); Sham (**b**); CCI (**c**); Pregabalin (**d**) Extract (200 mg/kg, p.o.) (**e**); Extract (400 mg/kg, p.o.) (**f**). Rats received different treatments for 14 days after which the animals were sacrificed, and tissues were collected. Arrowheads indicate myelin sheets degeneration and mononuclear infiltrating cells respectively. S; Schwann cell nuclei, E; exudate; hg, hemorrhage; Scale bar, 50 μm. Lower panel: quantification of myelin damage of sciatic nerves of different groups represented in figures (**a**–**f**) using Nerve Injury Scoring System (NISS), as follows: score 1, normal, mild degeneration or demyelination; 2, moderate level of degeneration or <50% damaged nerve tissue and 3, diffuse degeneration or demyelination > 50% damaged nerve tissue. * *p* < 0.05 vs. control values; # *p* < 0.05 vs. CCI group. Norm means normal group; Shm means Sham group; St 200 and St 400 mean *S. tetrasperma* extract (200 and 400 mg/kg) and pre means pregabalin group.

Hematoxylin and eosin (H&E) stained sections of sciatic nerves of both sham and control groups revealed well-organized myelin sheaths, absence of infiltrating cells and average number of Schwann cell nuclei (Figure 6a,b). On the other hand, stained nerve sections obtained from CCI rats showed multiple degenerations of myelin sheaths with hemorrhage and several areas of edema, mononuclear infiltrating cells and fewer Schwann cell nuclei (Figure 6c). Sections obtained from CCI rats treated with the extract (200 and 400 mg/kg, p.o.) showed a dose dependent improvement in the previous response with the higher dose showing restoration of most of the myelin sheath integrity and average Schwann cells numbers (Figure 6e,f). It was noted that the higher dose effect was comparable to that seen in sections obtained from pregabalin treated rats (Figure 6d).

#### 3.10.2. Effect on Brain Stem

**Figure 7:**
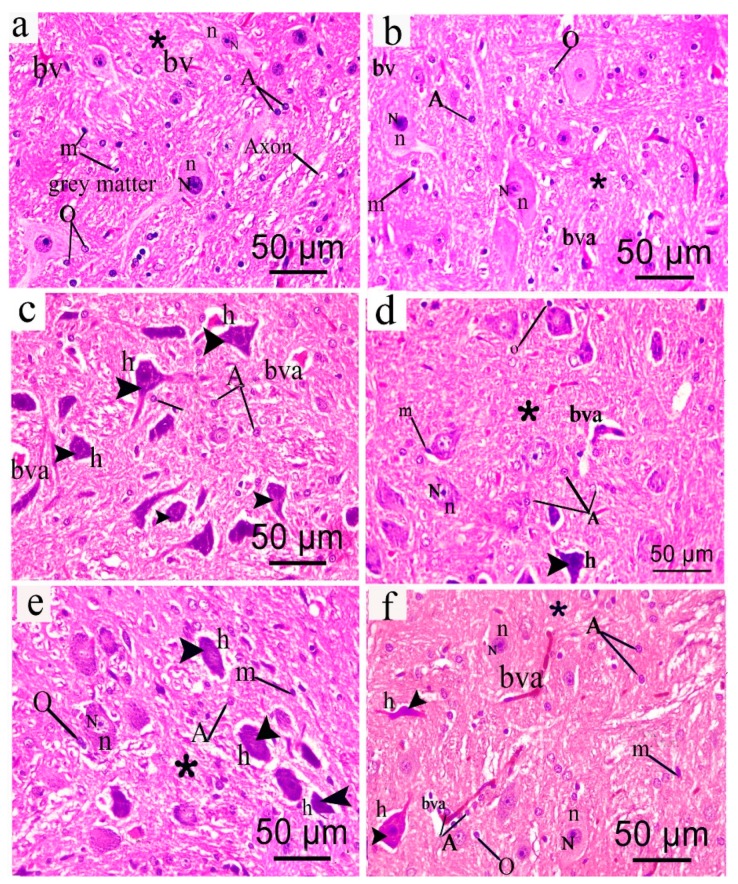
Representative photomicrograph (hematoxylin and eosin (H&E × 400) of brain stem sections from different groups: (**a**) Normal; (**b**) sham; (**c**) CCI (**d**) Pregabalin; (**e**) *S. tetrasperma* (200 mg/kg, p.o.); and (**f**) *S. tetrasperma* (400 mg/kg, p.o.). Arrowhead, arrow, bifid arrow, asterisk (*) illustrate normal neurons, affected neurons with dark stained cytoplasm and nuclei, perineural glial cell nuclei and neuropil, respectively. N; Nissl granule, n; nuclei, bv; normal blood vessel; bva, dilated congested blood vessel, h; halo, A; astrocyte. O; oligodentrites, m: microglial cells. Scale bar, 50 μm.

H&E stained brain stem sections of the control group revealed grey matter with normal neurons showing vesicular nuclei and basophilic cytoplasm having Nissl granules with long processes. The acidophilic neuropil (a dense network of interwoven nerve fibers and their branches and synapses, together with glial processes) showed supporting glial cells mainly astrocytes, oligodendrocytes and few microglial cells which had elongated nucleus. Normal blood vessels with narrow perivascular space (bv) were also seen (Figure 7a). The sham group showed the same structure of the control group but there were few affected neurons (Figure 7b). CCI sections revealed that most neurons were affected showing dark stained cytoplasm and nuclei surrounded by pericellular halo. The neuropil was also vacuolated indicating less glial cell number with congested blood vessel (Figure 7c). Sections obtained from rats treated with pregabalin showed some affected neurons with dilated congested blood vessel surrounded by perivascular halo (Figure 7d). In *S. tetrasperma* extract (200 mg/kg, p.o.) treated sections, most of the neurons showed to be affected along with congested blood vessels and only few neurons retained the normal structure (Figure 7e). However, treating rats with the higher extract dose (400 mg/kg, p.o.), showed that most neurons have retained normal architecture and demonstrated higher appearance of neuroglial cells compared to the CCI group (Figure 7f).

### 3.11. Behavioral and Neurological Toxicity Assessment

#### 3.11.1. Open Field Activity

**Figure 8:**
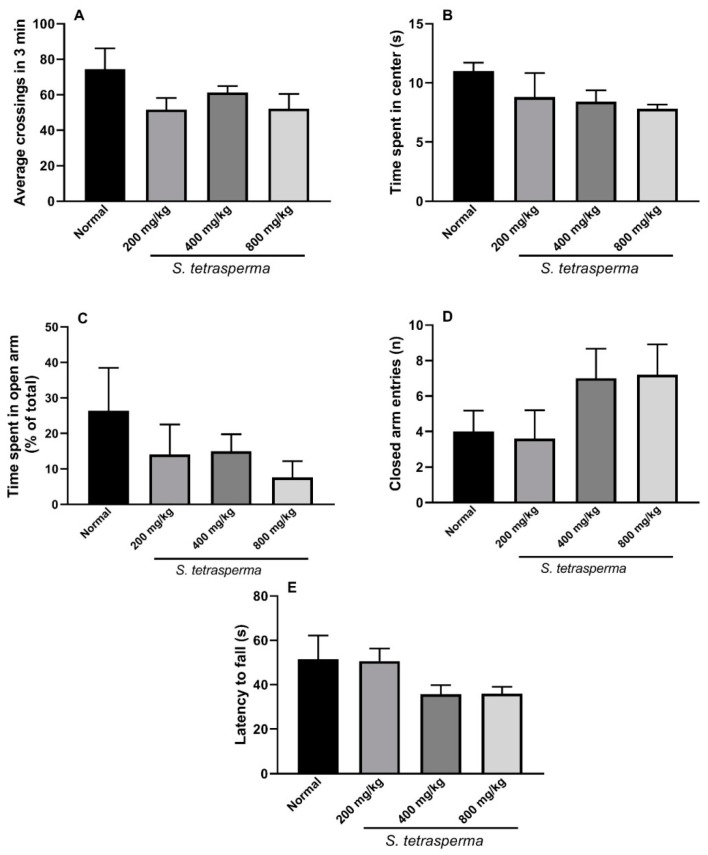
Effect of *S. tetrasperma* extract (200, 400 and 800 mg/kg, p.o) on: (**A**,**B**) open field activity total number of crossings and time spent in center, respectively; (**C**,**D**) elevated plus maze test time spent in open arm and number of closed arm entries, and (**E**) rotarod test latency to fall measurement. The values are expressed as the means ± SEM (*n* = 5–6).

As shown in Figure 8, mice treated with the extract (200, 400 and 400 mg/kg, p.o) tend to be less active as they show relatively lower number of crossings compared to untreated mice. Moreover, extract mice treated with high dose of the extract (400 and 800 mg/kg) show a trend to spend less time in center indicating a more anxious and less exploring reaction compared to control. However, the previous effects weren’t statistically significant.

#### 3.11.2. Emotionality

Mice receive the extract (200, 400 and 800 mg/kg, p.o) for 14 days, did not show any significant alteration in number of defecations compared to vehicle treated mice (0.20 ± 0.2, 0.80 ± 0.8 and 0.00 ± 0.0 vs. 0.20 ± 0.2), respectively. Similarly, urination frequency was not significant among different groups during the 3 min test period in the open field test, indicating that emotionality was not influenced by the extract.

#### 3.11.3. Elevated Plus Maze

There are no significant changes were obtained between different groups in the time spent in the open arm or the number of entries to the closed arm. Notably, there is a trend of mice receiving high doses of the extract (400 and 800 mg/kg) to spend less time in the open area and to have more entries to the closed arm indicating a mild anxious effect (Figure 8).

#### 3.11.4. Rotarod Test

In this test, rotarod apparatus was used to assess the effect of 14 days treatment of the extract (200, 400 and 800 mg/kg, p.o) on motor coordination and balance. As shown in Figure 8, there were no significant changes in latency to fall between the groups that received the vehicle or the extract at different doses. However, there was a tendency to a decrease in latency to fall with higher doses of the extract (400 and 800 mg/kg).

### 3.12. Investigation of Chronic Ulcerogenic Activity on Stomach

#### 3.12.1. Macroscopic Examination of Stomach

As shown in Table 4 and Figure S23, stomachs obtained from CCI rats showed small ulcer index (2.63). Treatment with the extract for 14 days resulted in a slight increase in the ulcer index with some (25%) of stomachs having minute ulcer spots. This mild side effect was intensified upon increasing the dose of the extract to 400 mg/kg p.o. demonstrated by increased ulcer index (5.88) and number of animals with ulcers (50%). Interestingly, the ulcer index of the extract (200 mg/kg, p.o.) is very close to that of the selective COX-2 inhibitor, celecoxib (3.31 vs. 2.4) and much less than that of the non-selective COX inhibitor, indomethacin which induced severe ulceration in stomachs and very high ulcer index (23.6) (Figure S23 and Table 4). This supports the SI data which reveals the extract’s selectivity to COX-2 (Table 4).

The ulcer index (UI) is the sum of average severity (US), average number (UN) and % lesion incidence (UP) according to the equation (UI = UN + US + UP × 10^−1^). Celecoxib (15 mg/kg, p.o.) and indomethacin (5 mg/kg, p.o.), were used as standards.

#### 3.12.2. Microscopic Examination of Rat Stomachs

The effect of the extract (200 and 400 mg/kg, p.o. for 14 days) treatment on both stomach and kidney architectures in CCI rats was investigated. The selective COX-2 inhibitor, celecoxib and the non-selective COX inhibitor, indomethacin were used as reference standards. Sections of the control stomach showed normal fundic mucosal layers consisting of epithelium, lamina propria and muscularis mucosa. The lamina propria has normal fundic glands that consists of isthmus, neck and base regions, opening into the surface by narrow gastric pits. Higher magnification of fundic mucosa revealed parietal cells with central rounded nuclei and eosinophilic cytoplasm (Figure S24a). CCI resulted in little shedding of gastric mucosa with submucosal edema, hemorrhage, slight mononuclear cell infiltration and few vacuolated parietal cells (Figure S24b). The low dose of the extract (200 mg/kg, p.o.) did not result in significant architectural changes compared to CCI sections and was comparable to the changes obtained with celecoxib treatment (Figure S24c,e). Noteworthy, the incidence of gastric side effects increased upon increasing the dose of the extract to 400 mg/kg represented as severe shedding in the fundic mucosa with sub mucosal edema and thick muscularis mucosa along with occurrence of some vacuolated parietal cells and little hemorrhage (Figure S24d). Indomethacin sections revealed erosion of the surface epithelial cells and loss of its normal architecture with edematous sub mucosal layer and mononuclear cell infiltration along with thick muscularis mucosa layer. Fundic glands were also dilated and the parietal cells had vacuolated cytoplasm and pyknotic nuclei (Figure S24f).

### 3.13. Effect on Kidneys

Control renal cortex sections stained with H&E revealed normal renal corpuscles, glomeruli and tubules (Figure S25a). In CCI group, there were few tubules with dark stained nuclei, some congested peritubular capillaries and congested glomerulus were seen (Figure S25b). Sections obtained from CCI rats treated with the extract in the lower dose (200 mg/kg, p.o.) revealed worsened histopathological picture compared to CCI group demonstrated as higher number of renal tubules with dark stained nuclei, congested and slightly shrunken glomerulus that are infiltrated with periglomerular cells (Figure S25c). The renal side effect seen with the extract grew worse upon increasing the dose to 400 mg/kg demonstrated by obvious renal tubules apoptosis and accumulation of exfoliated cells in the tubular lumen (Figure S25d).

### 3.14. Effect on the Liver

H&E stained liver sections of the control group revealed normal architecture of the liver hepatocytes with vesicular nuclei and basophilic cytoplasm radiating from normal central vein and separated by normal sinusoids which were lined by flat cells and contained Kupffer cells (Figure S26a). Sections from CCI group revealed congested central vein with slight cellular infiltrations with some hepatocytes showed dark stained nuclei and few congested sinusoids (Figure S26b). The *S. tetrasperma* (200 mg/kg, p.o.) treated group showed normal architecture of the liver hepatocytes radiating from slightly dilated central vein with some dilated congested sinusoids (Figure S26c). Sections obtained from rats treated with the higher extract dose (400 mg/kg) revealed markedly dilated and congested sinusoids, dilated central vein but with normal hepatocyte (Figure S26d).

## 4. Discussion

The major findings of the current study were as follows: (1) 38 secondary metabolites were characterized using LC-MS/MS. (2) The extract has antipyretic, analgesic and anti-inflammatory properties in all animal models tested (mice and rats). (3) Administration of the extract in CCI neuropathic pain model significantly improved neuropathic pain symptoms after thermal and mechanical induced hyperalgesia and allodynia. (4) The extract attenuated both NOX and iNOS enzymes and the associated oxidative and nitrosative stress in the CCI model. (5) The levels of the pro-inflammatory markers (NF-κB), cytokines (TNF-α), prostanoids (PGE2) and the enzymes (COX-2 and 5-LOX) showed a dose dependent decreased expression upon extract administration when measured in the sciatic nerve and the brain stem of CCI rats. (6) The extract is safe on stomach and liver, but it has considerable side effects on kidney which tend to increase upon increasing the dose.

The extract showed significant anti-inflammatory properties in all animal models and inhibited both early and late phases of inflammation. Similar activities were reported from the bark extract [6]. These activities directed our interest to further investigate the potential of the extract to replicate the same effects in a model of chronic pain and inflammation, namely the CCI model of neuropathy [8]. 

Utilizing a chronic model in the current study has more than one advantage. First, it highlights the potential of the extract to exert a beneficial effect when used in chronic inflammatory conditions. Second, it allows investigating the side effects of prolonged use on vital organs. Finally, it provides a better understanding of the molecular anti-inflammatory mechanisms involved in these effects. Our study clearly demonstrated that continuous administration of *S. tetrasperma* extract for 14 days after induction of neuropathic pain by CCI, effectively mediated the reversal of pain behaviors in the CCI model as it promoted recovery of tactile and thermal hypersensitivity when measured at 7, and 14 days post-surgery, suggesting a potential neuroprotective effect of the extract. The neuroprotective effect of the extract was compared with reference drug, pregabalin. Gabapentinoids like pregabalin and gabapentin represent a first-line treatment in chronic neuropathic pain and represent one of the most widely used drugs to control this condition. However, patients usually used higher doses for pain control which resulted in wide range of adverse effects [24].

The extract alleviated heat or thermal hyperalgesia, cold allodynia, mechanical allodynia and mechanical hyperalgesia by the two tested dose levels (200 and 400 mg/kg). This effect was comparable or even more potent than reference compound pregabalin. The extract not only improved neuropathic pain but also ameliorated the structural changes in both sciatic nerve and brain stem 14 days post-surgery.

The NADPH oxidase (NOX4) plays an important role in neuropathic pain development and its deficiency is associated with reduced CCI-induced up-regulation of sciatic nerve and dorsal root ganglia pro-inflammatory cytokines [25]. It was reported that overexpression of NOX and the associated oxidative stress is directly correlated with damage of sciatic nerve, while modulation of NOX can prevent oxidative damage in the rat model of sciatica [2]. These studies confirmed the association between the neuroprotective effect of the extract and the reduction of NOX expression observed in the current study. Several experimental studies have shown that ROS contribute to hyperalgesia and allodynia [26]. In the present study, the NOX level was attenuated while the level of the antioxidant catalase was increased in the sciatic nerve and brain stem indicating strong antioxidant potential of the extract. The extract also attenuated nitrosative stress markers via reducing iNOS expression in both sciatic nerve and brain stem. The superior result belonged to the 400 mg/kg dose of *S. tetrasperma* extract that was even better than the reference pregabalin. The anti-hyperalgesia status caused by the extract could be related to the significant decrease in the NO levels which is an important signal molecule in neuropathic pain, most likely through decreasing the iNOS expression in sciatic nerve and brain stem. It can be thus assumed that the inhibitory effects of the extract on pain behaviors in the rat CCI model, at least in part, are associated with its antioxidant activity. The antioxidant activity of the extract is attributed to the presence of diverse secondary metabolites such as protocatechuic acid, gallic acid, hydroxycinnamic acid derivatives (*p*-coumaric and caffeic acids) and the flavonoids (rutin and quercetin pentosyl-rutinoside).

It was observed, that CCI rats showed mononuclear infiltrating cells that release the pro-inflammatory cytokines (TNF-α and PGE2) which play a critical role in the pathogenesis of neuropathic pain. Interestingly, the pro-inflammatory cytokine, TNF-α can also exert a pro-apoptotic effect in different cell types [27]. It induces apoptosis through binding to TNF receptor (TNFR) in Schwann cells which explains the decreased number of Schwann cell nuclei in the CCI model animals observed in the current study [28]. The extract showed a dose dependent improvement in the previous response represented as a significantly lower degeneration score, with the higher dose showing restoration of most of the myelin sheath integrity and average Schwann cells number. The anti-inflammatory effects of the extract may be attributed to salicin and the salicylic acid derived from it. Also, catechol, the most important active metabolite of the common willow bark; the phenolic glucoside salicortin, may contribute to the anti-inflammatory activity [5].

A previous study performed by Gao et al., [29] used bioinformatics tools to identify the key genes in neuropathic pain and found that P53 is the key gene. This study also confirmed the results of bioinformatics study by measuring p53 in dorsal root ganglia following CCI. They reported that p53 is overexpressed in spinal cord and was associated with apoptosis of neurons and pretreatment with p53 inhibitor suppressed neuronal apoptosis. So, in our study we measured p53 in brain stem in CCI model, as a specific apoptotic marker for neuronal death associated with neuropathic pain. We found similar results to that showed by Gao et al., [29]. P53 is overexpressed in brain stem and caused neuronal apoptosis. The expression of the apoptotic p53 protein in CCI model was also investigated. It was reported previously that overexpression of p53 in neuronal cells is significantly linked to neuropathic pain and neurons degeneration [30]. Some synthetic p53 inhibitors were reported to protect against neurons death in strokes, ischemia, and neurodegenerative disorders. Most of these inhibitors chelate the Zn atom in the DNA binding domain of p53 which interferes with DNA transcription leading to denaturation and deactivation of the protein [31]. Our findings showed that CCI resulted in significantly higher levels of p53 in brain stems indicating more apoptotic and degenerative outcomes. The plant extract attenuated these changes in a dose dependent manner supporting its central neuroprotective effect. 

One possible mechanism that may underline the inhibitory effects of *S. tetrasperma* extract against neuropathic pain is the reduction of inflammatory cytokines and inflammatory enzymes. The extract inhibited both COX-2 and 5-LOX expression and their associated products such as PGE2 in sciatic nerve and brain stem. Furthermore, it suppressed the production of TNF-α and NF-κB which have a central role in the gradual expansion and maintenance of pain hypersensitivity induced by the nerve injury. In this study, both NF-κB and TNF-α rose markedly 14 days post-surgery, an effect that was significantly attenuated when rats were treated with the extract during this period.

Targeting a central component of neuropathic pain necessitates studying the potential of the extract to exert neurotoxic and/or behavioral untoward effects by exploring its ability to exert an anxiety-related behavior in mice utilizing two widely used behavioral tests in rodents, the open-field and the elevated-plus maze. Further, the effect of the extract on mice locomotor activity (number of crossings in open field test) and muscle coordination and balance (rotarod test), which indicates cerebellar dysfunction was also investigated [32]. Higher doses of the extract (400 and 800 mg/kg) showed a tendency to exert more anxious behavior in mice by decreasing the time spent in center in the open field and the time spent in the open arm while increasing the number of entries in the closed arm in the elevated plus maze test. Additionally, a trend to a decreased activity and motor coordination represented as relatively smaller number of crossings and shorter latency to fall time, respectively was also observed in the same doses. However, none of these changes were significant compared to vehicle treated controls (Figure 8). These findings suggest that, the extract lakes central toxicity when used at a dose of 200 mg/kg and is relatively safe when used at higher doses (400 and 800 mg/kg).

The main concern regarding long treatment with anti-inflammatory drugs is the risk of gastric and renal side effects, therefore, the effect of *S. tetrasperma* treatment on both the stomach and kidney architectures in CCI rats is investigated. Celecoxib (selective COX-2 inhibitor) and indomethacin (non-selective COX inhibitor) were used as reference standards. Both macroscopic and microscopic examination of rats’ stomachs showed that both dose levels of the extract have the least deleterious effect. The most aggressive agent was indomethacin. These findings are supported by the in vitro study which demonstrated the extract’s selective COX-2 inhibitory activity.

However, the lower dose (200 mg/kg) revealed worsened histopathological picture of kidney compared to the CCI group. This renal side effect aggravated upon increasing the dose to 400 mg/kg. Further studies are required to explain this effect on kidney. As for the liver, *S. tetrasperma* treated groups showed normal architecture with slightly dilated central vein and some congested sinusoids. 

The aforementioned activities of the extract might be attributed to its polyphenols content (among them flavonoids) where; similar studies have demonstrated that flavonoids such as rutin, quercetin, luteolin, hesperidin and bioflavonoids as well as polyphenols-rich extracts induce remarkable anti-nociceptive and/or anti-inflammatory activities [33,34,35,36,37].

## 5. Conclusions

Although *Salix* species and their main principle salicin were previously studied in different animal models, this is the first study to investigate the effect of a flower extract from *S. tetrasperma* on neuropathic pain model. The study highlights the extract’s putative molecular mechanism of action and its potential side effects as well. We showed that the extract has both peripheral and central analgesic and anti-inflammatory properties in addition to antipyretic effects. The extract attenuated CCI-induced thermal and mechanical pain behaviors in rats via combatting neuro-inflammation both peripherally and centrally where it showed to prohibit NF-κB, iNOS, NOX, COX-2 activation and prevent TNF-α, PGE2, p53 mediated neuronal death induced by peripheral nerves injury concluding that these biomarkers are the possible molecular targets for the extract components. The two dose levels of the extract showed to be relatively safe towards stomach and liver, however the low dose level showed to be associated with some renal risks.

## Figures and Tables

**Figure 1:**
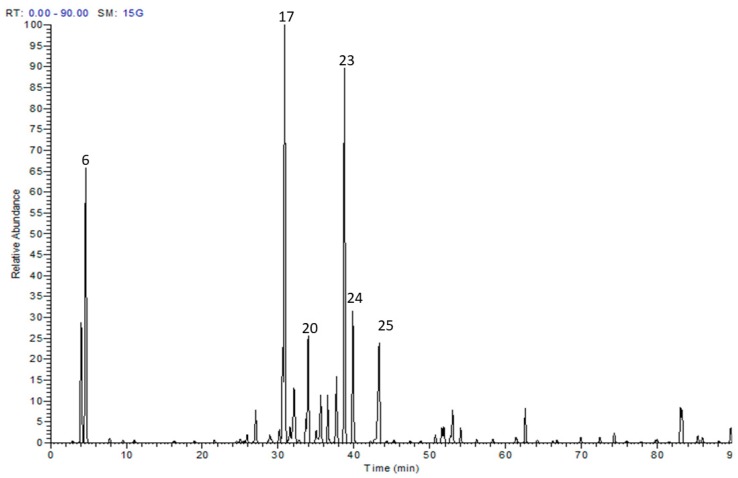
Liquid chromatography-mass spectrometry (LC-MS) base peak chromatogram of *Salix tetrasperma* flowers.

**Table 1:** Secondary metabolites from *Salix tetrasperma* flower extract.

No.	Rt	M–H	MS/MS	Tentatively Identified Compounds
1	2.49	315	153	Protocatechuic acid 3-*O*-hexoside
2	4.30	353	191, 179	Chlorogenic acid
3	4.72	295	133, 179	Caffeoylmalic acid
4	4.98	401	123, 285, 383	Salicin malate
5	6.39	285	123	Salicin
6	7.01	337	163	Coumaroylquinic acid
7	7.73	279	163	Coumaroyl malic acid
8	9.35	165	119, 147	Phloretic acid
9	16.13	385	179, 223	Sinapic acid 3-*O*-glucoside
10	16.30	625	179, 301, 463	Quercetin dihexoside
11	18.64	327	123	2′-*O*-acetyl-salicin
12	21.90	477	169, 331	Coumaroylgalloyl glucose
13	24.34	741	301, 591, 609	Quercetin pentosyl-rutinoside
14	24.76	423	161, 285	Salicortin
15	25.92	595	179, 301, 463	Quercetin pentosyl-hexoside
16	29.02	755	315, 623	Isorhamentin pentosyl-rutinoside
17	31.02	609	179, 301	Rutin
18	31.54	463	151, 179, 463	Quercetin 3-*O*-glucoside
19	32.08	609	315, 459, 477	Isorhamentin pentosyl hexoside
20	33.62	447	179, 285	Kaempferol 3-*O*-glucoside
21	35.11	447	179, 285	Kaempferol 3-*O*-glactoside
22	36.61	477	151, 179, 315	Isorhamentin 3-*O*-glucoside
23	37.89	431	145, 163, 307	Trichocarposide
24	40.11	461	193, 299,	Kaempferide 3-*O*-hexoside
25	42.12	461	315	Isorhamentin 3-*O*-rhamnoside
26	44.26	417	145, 163, 307	Dihydrocinnamoyl salicin
27	48.32	415	145, 163, 307	Cinnamoyl salicin
28	54.04	527	155, 405	Tremulacin
29	56.17	577	269	Apigenin coumaroyl-glucoside (Terniflorin)
30	61.10	269	269	Apigenin
31	62.59	299	151, 284, 299	Kaempferide
32	66.22	569	163, 307, 423, 431	Coumaroyl dihydrobenzoyl salicin
33	74.36	569	163, 307, 423, 431	Coumaroyl dihydrobenzoyl salicin
34	79.95	295	171, 277, 295	Hydroxy octadecadienoic acid
35	81.49	293	171, 275, 293	Hydroxy-octadecatrienoic acid
36	83.11	843	455, 559	Oleanolic acid derivative
37	83.49	855	413, 575, 855	Sitosterol glucoside linoleic acid
38	89.63	861	419, 581, 861	Acutifoliside glucoside linoleic acid

**Table 2:** In vitro cyclooxygenase-1 (COX-1), cyclooxygenase-2 (COX-2) and lipoxygenase (LOX) inhibition (IC_50_) and the total antioxidant capacity (U/L) of the *S. tetrasperma* extract.

Treatment	COX-1	COX-2	SI	5-LOX	TAC
	IC_50_ (µg/mL)			IC_50_ (µg/mL)	U/L
Extract	10.07 ± 0.55	0.089 * ± 0.01	113.1 *	3.86 ± 0.22	30.97 ± 2.6
Celecoxib	15.63 ± 1.2	0.056 ± 0.01	279.1	-	-
Diclofenac	4.13 ± 0.5	0.79 ± 0.13	5.23	2.6 ± 0.44	-
Indomethacin	0.09 ± 0.006	0.73 ± 0.1	0.12	-	-
Zileuton	-	-	-	3.17 ± 0.32	-
Ascorbic acid	-	-	-	-	26.8 ± 2.1

SI is COX selectivity index which is defined as IC_50_ (COX-1)/IC_50_ (COX-2). Values are mean ± S.E.M. * *p* < 0.05 vs. diclofenac and indomethacin values. TAC—total antioxidant capacity.

**Table 3:** Effect of *S. tetrasperma* extract on Brewer’s yeast induced pyrexia in mice.

Experiment	Dose (mg/kg)	Rectal Temperature ^@^	Rectal Temperature Recorded Following Different Treatments				
			30 min	1 h	2 h	3 h	24 h
Control	-	37.8 ± 0.42	38.52 ± 0.10	38.5 ± 0.12	39.05 ± 0.28	39.00 ± 0.34	38.28 ± 0.19
Extract	200	39.02 ± 0.29	37.23 ± 0.42 *	38.25 ± 0.49	38.52 ± 0.5	38.13 ± 0.23	37.88 ± 0.39
Extract	400	38.72 ± 0.29	37.22 ± 0.22 *	38.7 ± 0.2	38.28 ± 0.21	37.92 ± 0.22 *	37.34 ± 0.08
Paracetamol	150	38.56 ± 0.19	38.14 ± 0.19	37.56 ± 0.30	37.04 ± 0.29 *	36.9 ± 0.25 *	36.38 ± 0.22 *

^@^ Rectal temperature 18 h after yeast injection. Values are expressed as mean ± S.E.M (*n* = 5), * *p* < 0.001 vs. control values.

**Table 4:** Chronic ulcerogenecity of *S. tetrasperma* extract as compared to two reference drugs celecoxib and indomethacin.

Treatment	Average Ulcer Number	Average Severity Score	Lesion Incidence	Ulcer Index
	(UN)	(US)	(UP, %)	(UI)
Control (CCI)	0.25	0.125	20	2.63
Indomethacin	12.6	1.49	100	23.6
Celecoxib	0.2	0.2	20	2.4
Extract (200 mg/kg, p.o.)	0.5	0.31	25	3.31
Extract (200 mg/kg, p.o.)	0.5	0.375	50	5.88

## Supplementary Materials

The following are available online at https://www.mdpi.com/2076-3921/8/10/482/s1, Table S1. Secondary metabolites from *Salix tetrasperma* flower extract. Figure S1. A photo of *S. tetrapserma* flowers. Figure S2. MS/MS profile of protocatechuic acid 3-O-hexoside. Figure S3. MS/MS profile of chlorogenic acid. Figure S4. MS/MS profile of caffeoylmalic acid. Figure S5. MS/MS profile of salicin malate. Figure S6. MS/MS profile of salicin [M − H + 46, formic acid adduct]. Figure S7. MS/MS profile of coumaroylquinic acid. Figure S8. MS/MS profile of coumaroylmalic acid. Figure S9. MS/MS profile of phloretic acid. Figure S10. MS/MS profile of sinapic acid 3-O-hexoside [M − H + 46, formic acid adduct]. Figure S11. MS/MS profile of 2′-*O*-acetyl-salicin [M − H + 46, formic acid adduct]. Figure S12. MS/MS profile of coumaroylgalloyl glucose. Figure S13. MS/MS profile of salicortin [M − H + 46, formic acid adduct]. Figure S14. MS/MS profile of rutin. Figure S15. MS/MS profile of trichocarposide. Figure S16. MS/MS profile of dihydrocinnamoyl salicin. Figure S17. MS/MS profile of cinnamoyl salicin. Figure S18. MS/MS profile of coumaroyl dihydrobenzoyl salicin. Figure S19. Effect of 1 h prior administration of *S. tetrasperma* (200, 400 and 600 mg/kg, p.o.). diclofenac sodium (20 mg/kg, p.o) or dexamethasone (2 mg/kg, p.o) on carrageenan (1% suspension, 0.1 mL/rat) induced-hind paw edema in rats. Edema thickness (mm) was measured before and then hourly for 5 h and at 24 h after carrageenan injection. Data represents the AUC0-24 and is expressed as mean ± S.E.M (*n* = 5). * *p* < 0.05 vs. control values. Figure S20. Effect of carrageenan (500 µg, ip) on leukocytes migration into the peritoneal cavity in mice (total number ×106) with or without 1 h prior treatment with *S. tetrasperma* (200, 400 and 600 mg/kg, p.o.), diclofenac sodium (20 mg/kg, p.o) or dexamethasone (2 mg/kg, p.o). Data is expressed as mean ± S.E.M (*n* = 5–7). * *p* < 0.01 vs. vehicle (saline) values, # *p* < 0.01 vs. control (carrageenan treated group). Figure S21. Effect of *S. tetrasperma* extract (200, 400 and 600 mg/kg, p.o.) on acetic acid-induced vascular permeability. The amount of Evans blue dye in the abdominal cavity was measured as an indicator of inflammation degree. The values are expressed as the means ± SEM (*n* = 5–6). * *p* < 0.001 compared to saline group. # *p* < 0.001 compared to control (acetic acid only treated group). Figure S22. Effect of *S. tetrasperma* (200 and 400 mg/kg, p.o.), diclofenac (20 mg/kg, p.o) or dexamethasone (2 mg/kg, p.o) on acetic acid-induced writhing (0.7%, 1 mL/100 g) in mice (A). Hot plate response latency (s) measured 1–4 h after vehicle, the extract (200 and 400 mg/kg, p.o.) or nabuphine (10 mg/kg, p.o) administration in mice (B). Data is expressed as mean ± S.E.M (*n* = 5–8). * *p* < 0.001 vs. control values. Figure S23. Representative stomach images obtained from CCI rats receiving either the vehicle or different treatments. Figure S24. Representative photomicrograph of stomach sections (fundic mucosa) from different groups, (a) control; (b) CCI; (c) celecoxib; (d) indomethacin; (e) *S. tetrasperma* (200 mg/kg) and (f) *S. tetrasperma* (400 mg/kg). The arrow heads, double arrow, arrow, curved arrows, thin bifid arrow, thick bifid arrows and twisted arrows, short arrow, thick arrow and astrexic illustrate normal fundic mucosa, normal fundic glands, wide fundic glands, normal parietal cell, remnant of cytoplasm of parital cells, dark stained nuclei of parital cell, glandular cells with karyolitic nuclei, slight shedingfundic mucosa, erosion of fundic mucosa and gastric pit respectively. Isthmus (I), N (neck), basic region (B), mucosa (M), muscularis mucosa (Ms), serosa (S), edema (E), infilteration (If), hemorrhage (hg), vacuoles (v) and blood vessel (bv) Scale bar, 200 μm × 100, 50 μm × 400. Figure S25. Representative photograph of renal cortex sections of the different groups. (a) control; (b) CCI; (c) S. tetrasperma (200 mg/kg); (d) *S. tetrasperma* (400 mg/kg). Arrow and arrow head illustrate the dark stained nuclei and infilterating cells respectively. G, glomerulus; Cg, congested glomeruli; PT, proximal convoluted tubule; DT, distal convoluted tubule; T, affected tubule; E, exfoliated cells; C, peritubular capillaries. Figure S26. Reprehensive photograph in sections of liver from different groups, (a) control; (b) CCI; (c) *S. tetrasperma* (200 mg/kg); (d) *S. tetrasperma* (400 mg/kg). Arrowhead, curved arrow and twisted arrow illustrate normal hepatocytes, Kupffer cells, hepatocytes with dark stained nuclei infiltrations respectively. S, the blood sinusoids; Cv, the central vein, Scale bar, 50 μm × 400.

## Abbreviations

CCI: chronic constriction induced sciatic nerve injury, COX-2; cyclooxygenase-2, HPLC-MS/MS; High performance liquid chromatography mass spectrometry, iNOS; Inducible nitric oxide synthase, 5-LOX; 5-lipoxygenase, NOX1; Nicotinamide adenine dinucleotide phosphate (NADPH) oxidase 1, NF-κB; Nuclear factor kappa-light-chain-enhancer of activated B cells and PGE2; prostaglandin E2, TNF-α; Tumor necrosis factor-α.
