# Supplementary material for: Salix tetrasperma Roxb. Extract Alleviates Neuropathic Pain in Rats via Modulation of the NF-κB/TNF-α/NOX/iNOS Pathway
Source: Antioxidants (Basel). 2019 Oct 12;8(10):482. doi: 10.3390/antiox8100482 (PMC6826723; doi:10.3390/antiox8100482)
Supplement: Supplementary file 1 [file antioxidants-08-00482-s001.pdf]

***Salix tetrasperma* Roxb. extract alleviates neuropathic pain in rats *via* modulation of NF-κB/TNF-α/NOX/iNOS pathway**

Materials and methods

HPLC-MS analysis

A ThermoFinnigan LCQ-Duo ion trap mass spectrometer (ThermoElectron Corporation, Waltham, Ma, USA) with an Electrospray ionization (ESI) source (ThermoQuest Corporation, Austin, Tx, USA) was used. A Discovery HS F5 column (15 cm x 4.6 mm ID, 5 µm particles, Sigma-Aldrich Co Steinheim, Germany) was utilized with the ThermoFinnigan HPLC system. The mobile phase was water and acetonitrile (ACN) (Sigma-Aldrich GmbH, Steinheim, Germany) (0.1 % formic acid each). At 0 min, ACN was 5%, then increased to 30% over 60 min, and finally to 90% within the last 30 min. The flow rate was kept at 1 mL/min with a 1:1 split before the ESI source. The MS operated in the negative mode as previously reported (Ghareeb et al. 2018).

In vitro antioxidant activities

The total antioxidant capacity (TAC) assay was investigated using the commercially available TAC ELISA kit (MBS726896, my BioSource, Inc., San Diego, CA, USA) following the manufacturer's instructions as Cyclooxygenase (COX) and lipoxygenase (LOX) inhibition assays

The potential inhibition of ovine COX-1 and COX-2 by the extract was evaluated with an enzyme immuno assay (EIA) kit (Cayman Chemical, AnnArbor, MI, USA) as per manufacturer's instructions and previous reports (Abdelall, Lamie et al. 2016). To study the inhibition of lipoxygenase we used the screening assay kit (Cayman Chemical, AnnArbor, MI, USA) as per manufacturer's instructions and previous reports (Abdelall, Lamie et al. 2016).

Carrageenan-induced hind-paw edema

Freshly prepared carrageenan solution (1% in 0.9% NaCl, 0.1 mL) was injected into the sub plantar tissues of the right hind paw of rats to induce the edema. One hour earlier, the extract (200, 400 and 600 mg/kg, p.o.), diclofenac (10 mg/kg), or vehicle (10 mL/kg) were orally applied to the rats. A caliper ruler was used to measure the paw thickness (mm) in the dorsal-plantar axis before and after carrageenan injection as previously described (Sobeh, Mahmoud et al. 2018).

Recruitment of leukocyte to peritoneal cavity in mice

Swiss albino mice (n=5-8/ group) were treated with the extract (200, 400 and 600 mg/kg, p.o.) or the vehicle (1 mL/100 g, p.o.) 30 min before either 0.1 mL carrageenan solution (500 μg/mice) or 0.1 mL sterile saline were injected (i.p.). The experiments were carried out as previously described (Sobeh, Mahmoud et al. 2018).

Acetic acid-induced vascular permeability

The acetic acid-induced vascular permeability was performed as previously described (Lee, Choo et al. 2009). Briefly, mice treated with the extract (200 and 400 and 600 mg/kg, p.o.), diclofenac (20 mg/kg) or vehicle. 1 h. later 0.2 mL Evans blue (0.25% solution in normal saline) was injected in the tail vein. After 30 min, acetic acid (0.6% in normal saline, 1 mL/100g) was injected in the peritoneal cavity of mice. Another group of mice was injected with normal saline only and served as normal control. After another 30 min, the mice were sacrificed by cervical dislocation. The abdominal cavities were washed with 3 mL saline and the washings were then centrifuged at 3000 rpm for 10 min. The vascular permeability is proportional to Evans blue dye content of the supernatant that was detected at 610 nm using a plate reader (BioTeK, Vt, USA).

Anti-nociceptive activity

Acetic acid-induced abdominal writhing and the hot plate test were carried out as described in our previous work (Sobeh, Mahmoud et al. 2018).

Induction of fever in mice using Brewer's yeast

To study a potential antipyretic effect in mice we employed the Brewer's yeast model of pyrexia as previously described (Sengar, Joshi et al. 2015; Liu, Su et al. 2017) with some modifications. After recording the rectal temperature of mice with a lubricated digital thermometer, a 30% Brewer's yeast suspension in normal saline was injected subcutaneously (1 mL/100 g). After 18 h, rectal temperatures were measured (T_0) again. Only animals with at least 0.5°C higher temperature were included in the test. These mice were treated with 200 and 400 mg extract/kg, paracetamol (150 mg/kg) or vehicle. Again, the rectal temperature was measured at 30 min, 1, 2, 3 and 24 h after treatment.

Results

The secondary metabolites were identified based on their molecular weights, fragmentation pattern, retention times, available literature and authentic compounds (**Table S1**). A photo of the flowers is shown in **Figure S1.** The fragmentation pattern of some selected compounds in the flower extract is documented below (**Figures S2–S18**).

Table S1. Secondary metabolites from *Salix tetrasperma* flower extract

No.	Tentatively identified compounds	Previous identification	Reference
1	Protocatechuic acid-*O*-hexoside*	*S. tetrasperma*	El-Wakil et al., 2015
2	Chlorogenic acid**	*S. alba*<br>*S. amplexicaulis*<br>*S. babylonica*<br>*S. eleagnos*<br>*S. fragilis*<br>*S. purpurea*<br>*S. triandra*	Gligorić et al., 2019
3	Caffeoylmalic acid***	-	Pobłocka-Olech et al., 2010
4	Salicin malate***		
5	Salicin*	*S. tetrapserma*<br>*S. alba*<br>*S. Americana*<br>*S. cv. Aquatica*<br>*S. arbusculoides*<br>*S. aurita*<br>*S. babylonica*<br>*S. callicarpa*<br>*S. alba*<br>*S. amplexicaulis*<br>*S. babylonica*<br>*S. eleagnos*<br>*S. fragilis*<br>*S. purpurea*<br>*S. triandra*	El-Shazly et al., 2012<br>Boeckler et al. 2011<br>Gligorić et al., 2019
6	Coumaroylquinic acid*	*S. tetrasperma*	El-Wakil et al., 2015
7	Coumaroyl malic acid	-	Csernatoni et al., 2014
8	Phloretic acid***	-	Tanagornmeatar et al., 2014
9	Sinapic acid 3-*O*- glucoside***	-	Nićiforović and Abramovič, 2014
10	Quercetin dihexoside	*S. myrsinifolia Salisb.*	Nybakken and Julkunen-Tiitto, 2013
11	2'-*O*-Acetyl-salicin	*S. lasiandra*<br>*S. glandulosa*	Boeckler et al. 2011; Kim et al., 2015
12	Coumaroylgalloyl glucose***		
13	Quercetin pentosyl-rutinoside***		Barros et al., 2012

14	Salicortin	*S. alba*<br>*S. Americana*<br>*S. cv. Aquatica*<br>*S. arbusculoides*<br>*S. aurita*<br>*S. babylonica*<br>*S. callicarp*	Boeckler et al. 2011
15	Quercetin pentosyl-hexoside***		Bakour et al., 2019
16	Isorhamentin pentosyl-rutinoside***		
17	Rutin	*S. chaenomeloides*<br>*S. alba*<br>*S. amplexicaulis*<br>*S. babylonica*<br>*S. eleagnos*<br>*S. fragilis*<br>*S. purpurea*<br>*S. triandra*	Mizuno et al. 1991<br>Gligorić et al., 2019
18	Quercetin 3-*O*-glucoside	*S. chaenomeloides*<br>*S. raddeana*	Mizuno et al. 1991<br>Xu et al., 2007
19	Isorhamentin pentosyl hexoside***	-	Wang et al., 2012
20	Kaempferol 3-*O*-glucoside	*S. raddeana*	Xu et al., 2007
21	Kaempferol 3-*O*-galactoside***	-	Löffler et al., 1997
22	Isorhamentin 3-*O*-glucoside	*S. chaenomeloides*	Mizuno et al. 1991
23	Trichocarposide	*S. martiana*	Boeckler et al. 2011
24	Kaempferide 3-*O*-hexoside***	-	
25	Isorhamentin 3-*O*-rhamnoside***	-	Sobral et al., 2017
26	Dihydrocinnamoyl salicin***	-	
27	Cinnamoyl salicin	-	Kumari et al., 2016<br>Keefover-Ring et al., 2014
28	Tremulacin*	*S. tetrapserma*<br>*S. petiolaris*<br>*S. triandra*	El-Shazly et al., 2012<br>Boeckler et al. 2011
29	Apigenin coumaroyl-glucoside (Terniflorin)	*S. alba*	Ahmad et al., 1984
30	Apigenin	*S. lindeyana*	Thapliyal and Bahuguna et al., 1993
31	Kaempferide***		Przybyłek and Karpiński et al., 2019
32	Coumaroyl dihydrobenzoyl salicin***		
33	Coumaroyl dihydrobenzoyl salicin isomer***		
34	Hydroxy-octadecadienoic acid***		
35	Hydroxy-octadecatrienoic acid***		Chechetkin et al., 2019
36	Oleanolic acid derivative***		
37	Sitosterol glucoside linoleic acid***		
38	Acutifoliside glucoside linoleic acid***		

*Previously isolated from *S. tetrasperma* leaves

**Compound confirmed using a reference compound

***New compounds in genus *Salix* (Tentatively identified)

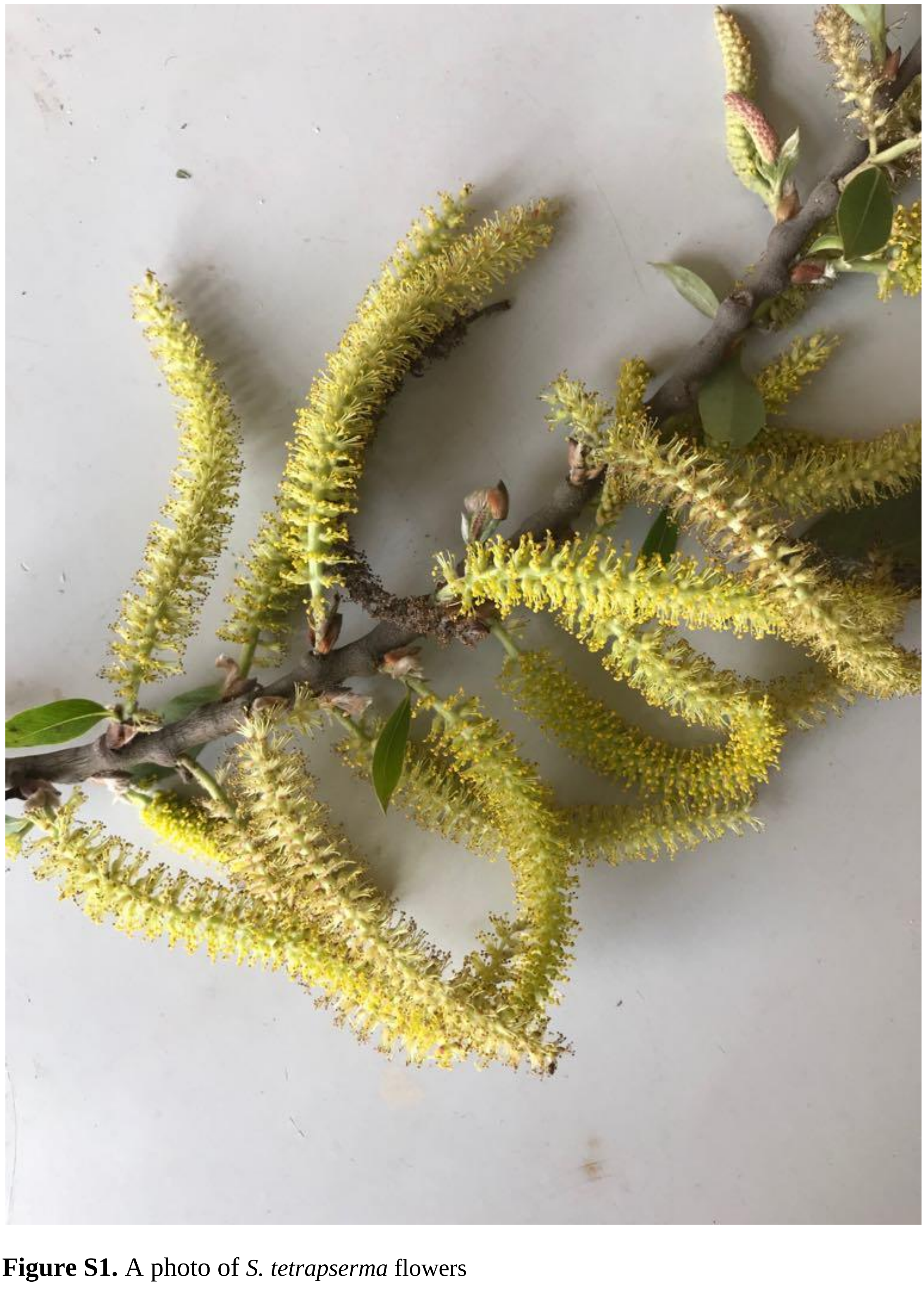

Figure S1. A photo of *S. tetrapserma* flowers

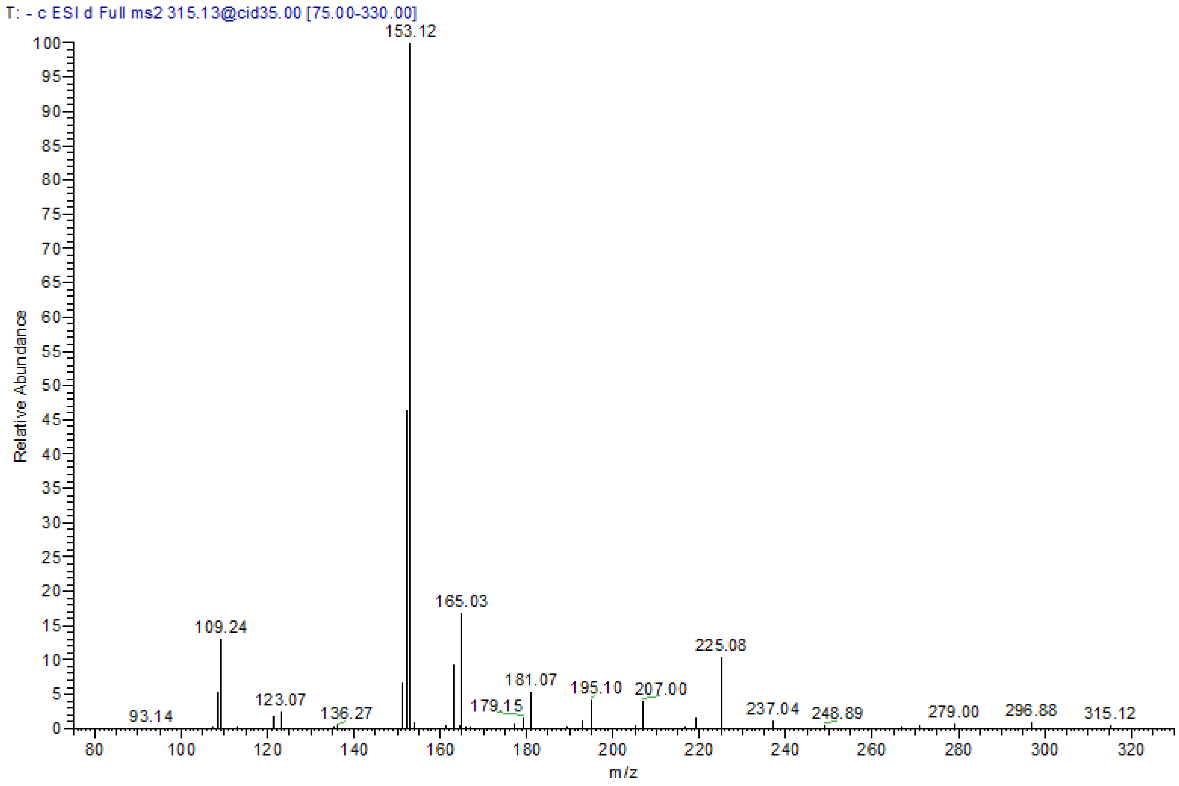

Figure S2. MS/MS profile of protocatechuic acid 3-*O*-hexoside

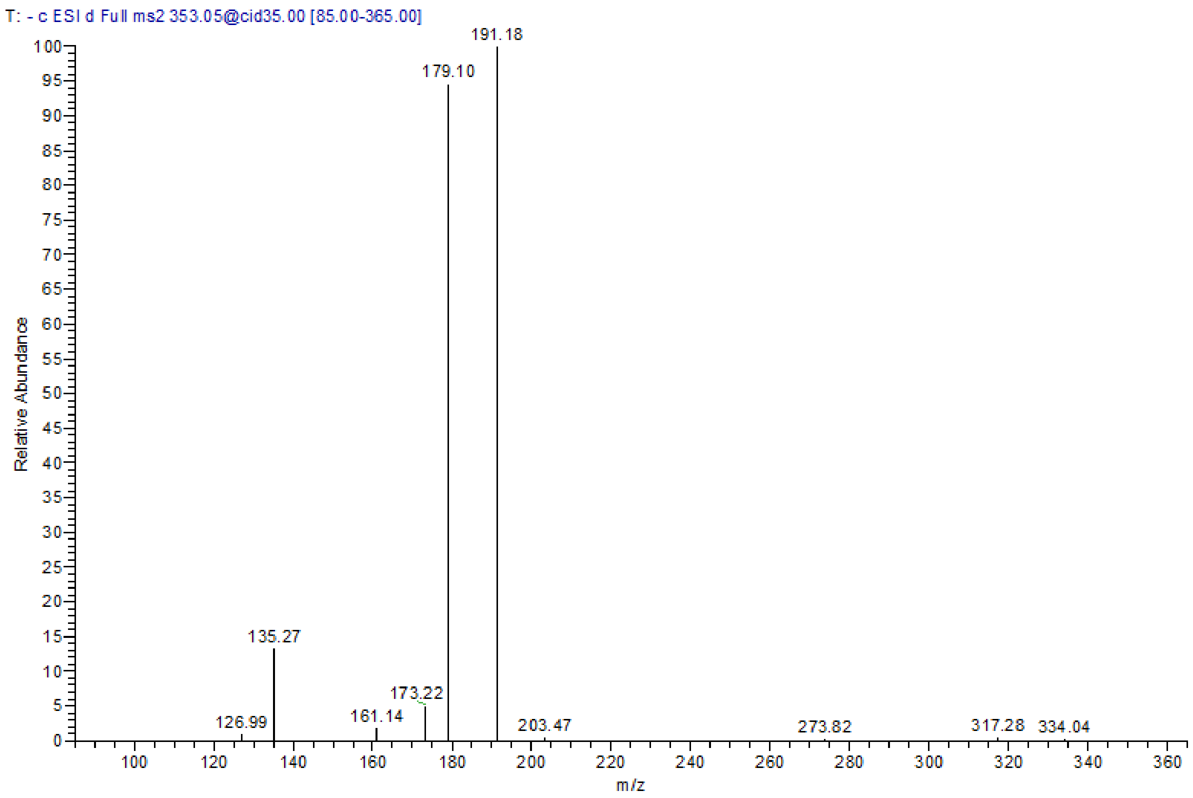

Figure S3. MS/MS profile of chlorogenic acid

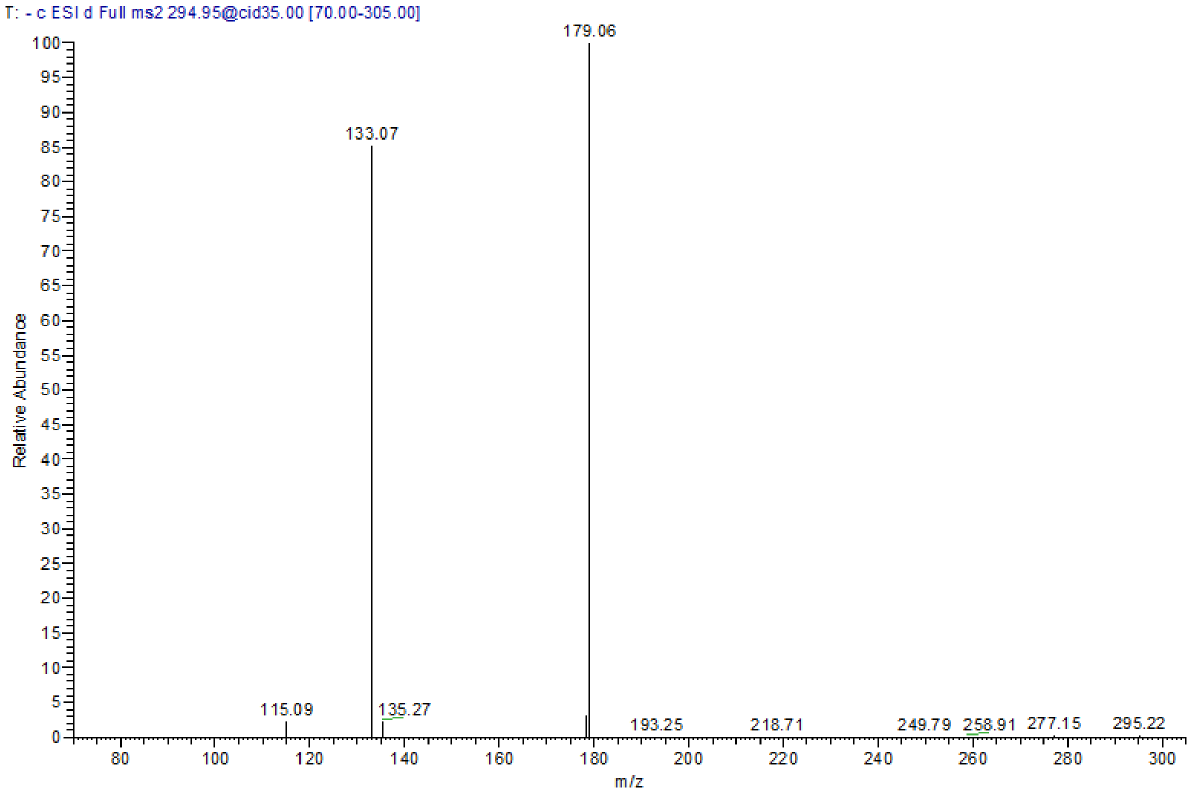

Figure S4. MS/MS profile of caffeoylmalic acid

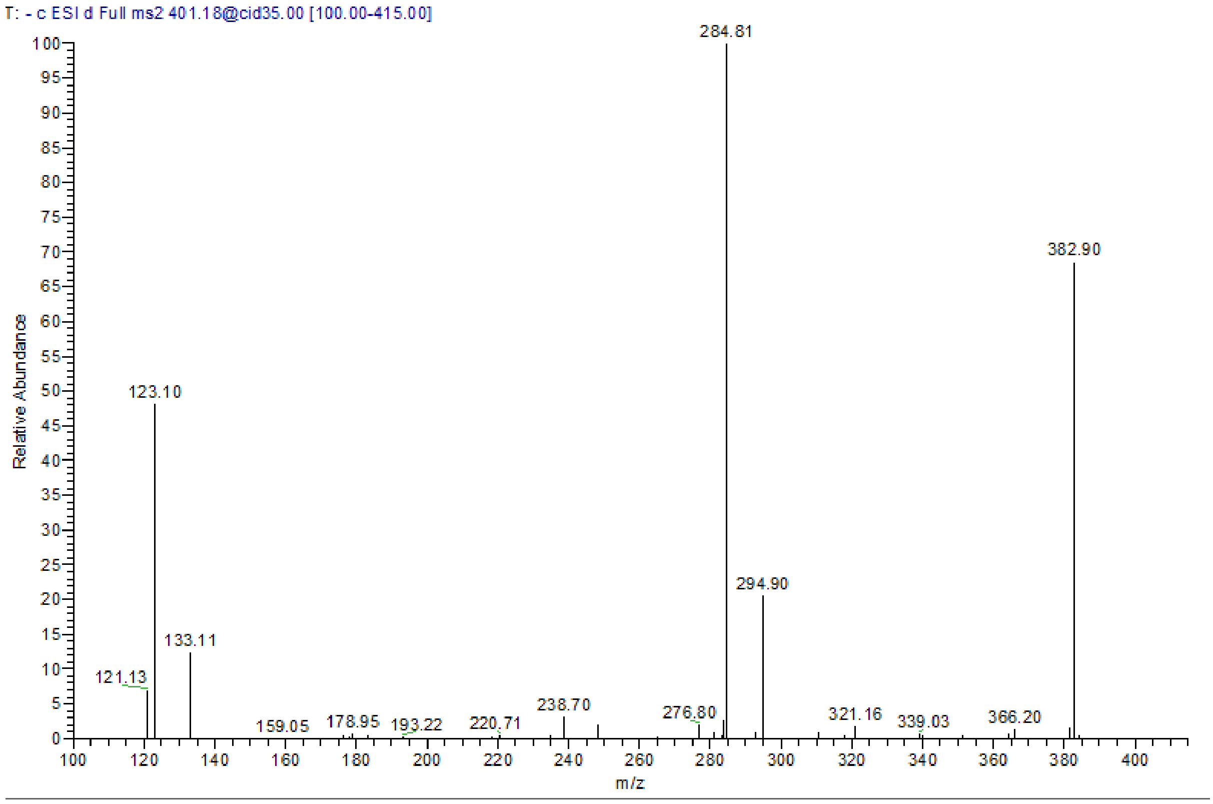

Figure S5. MS/MS profile of salicin malate

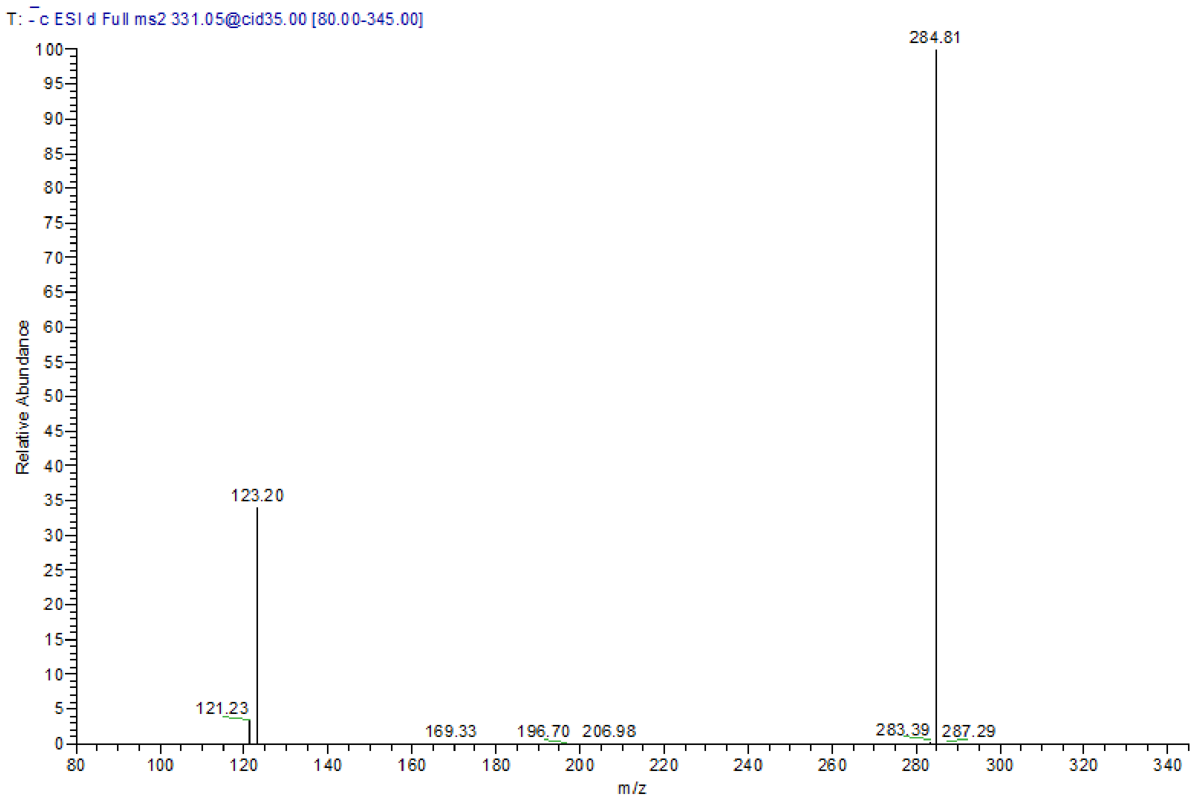

Figure S6. MS/MS profile of salicin [M-H + 46, formic acid adduct]

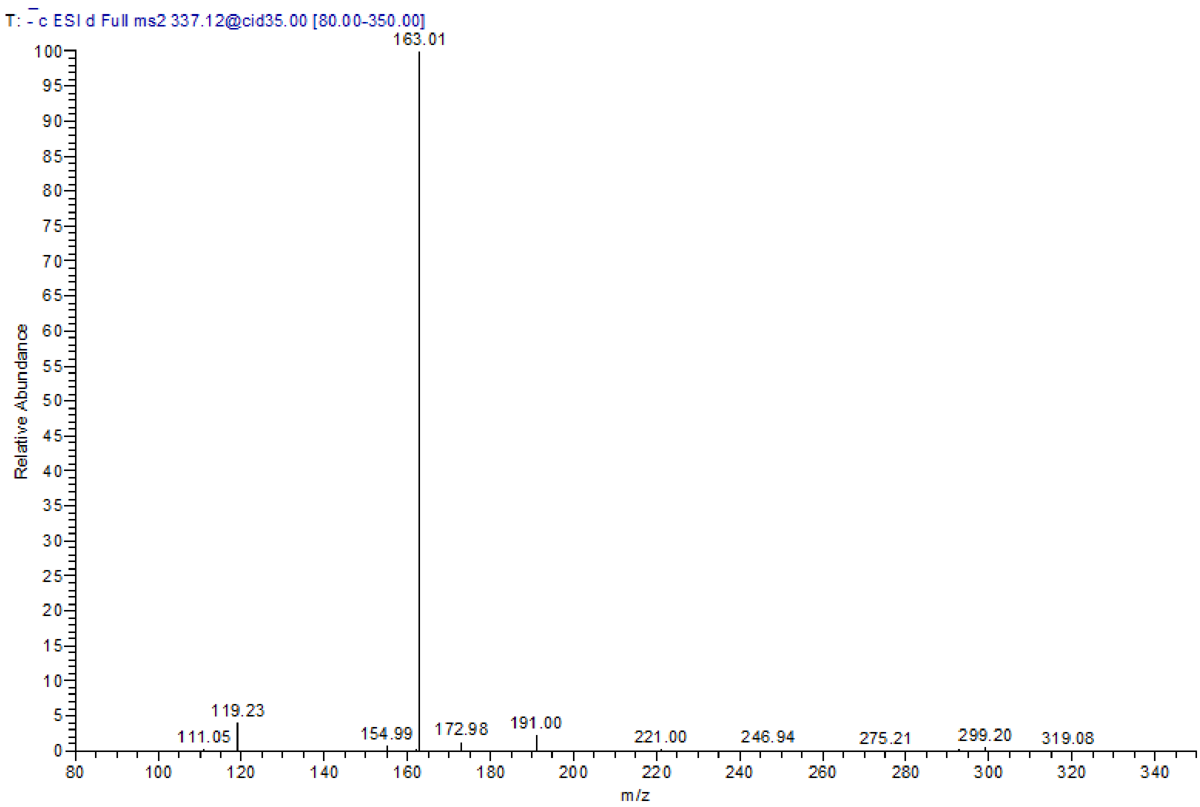

Figure S7. MS/MS profile of coumaroylquinic acid

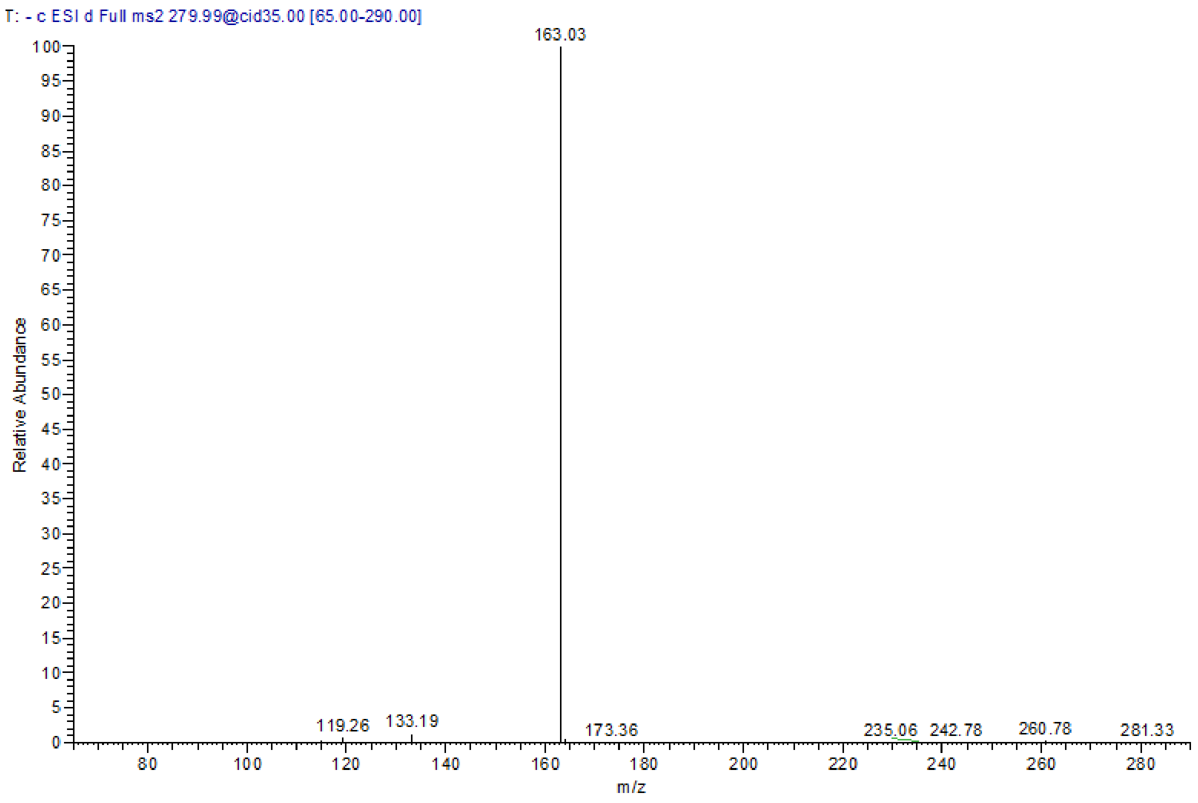

Figure S8. MS/MS profile of coumaroylmalic acid

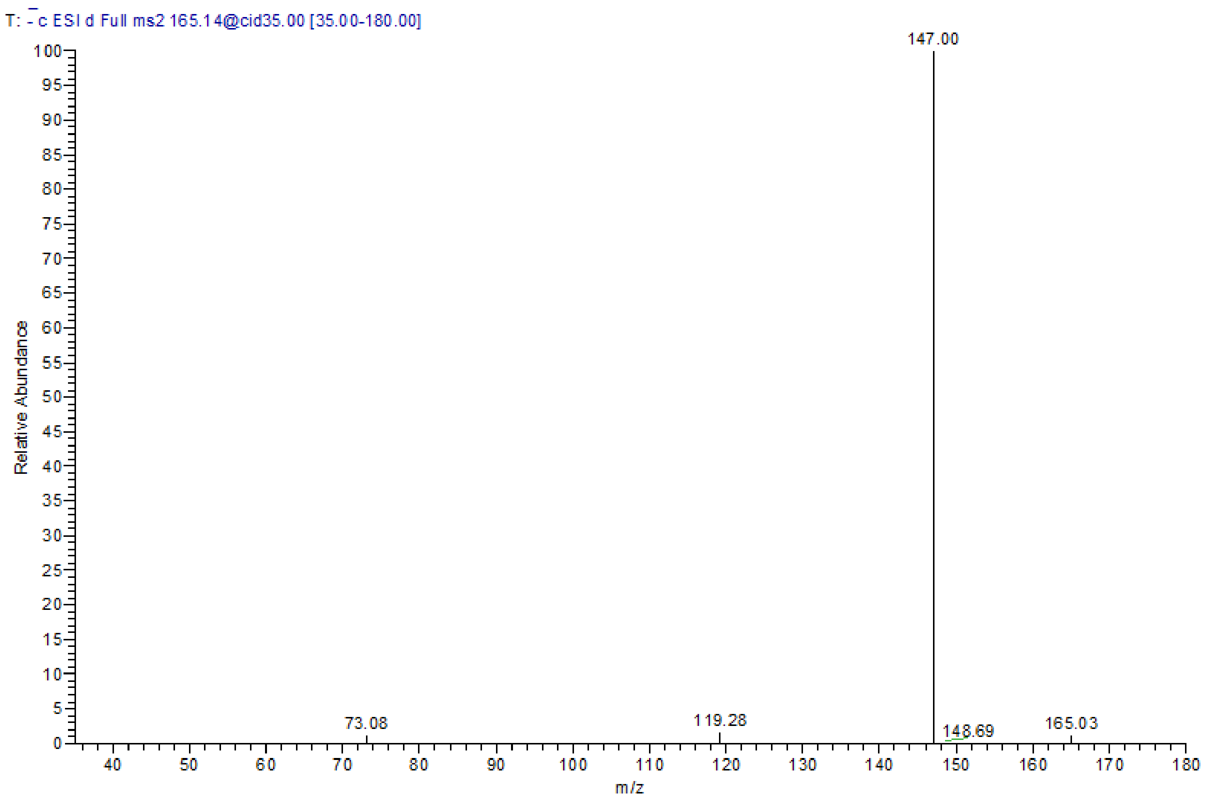

Figure S9. MS/MS profile of phloretic acid

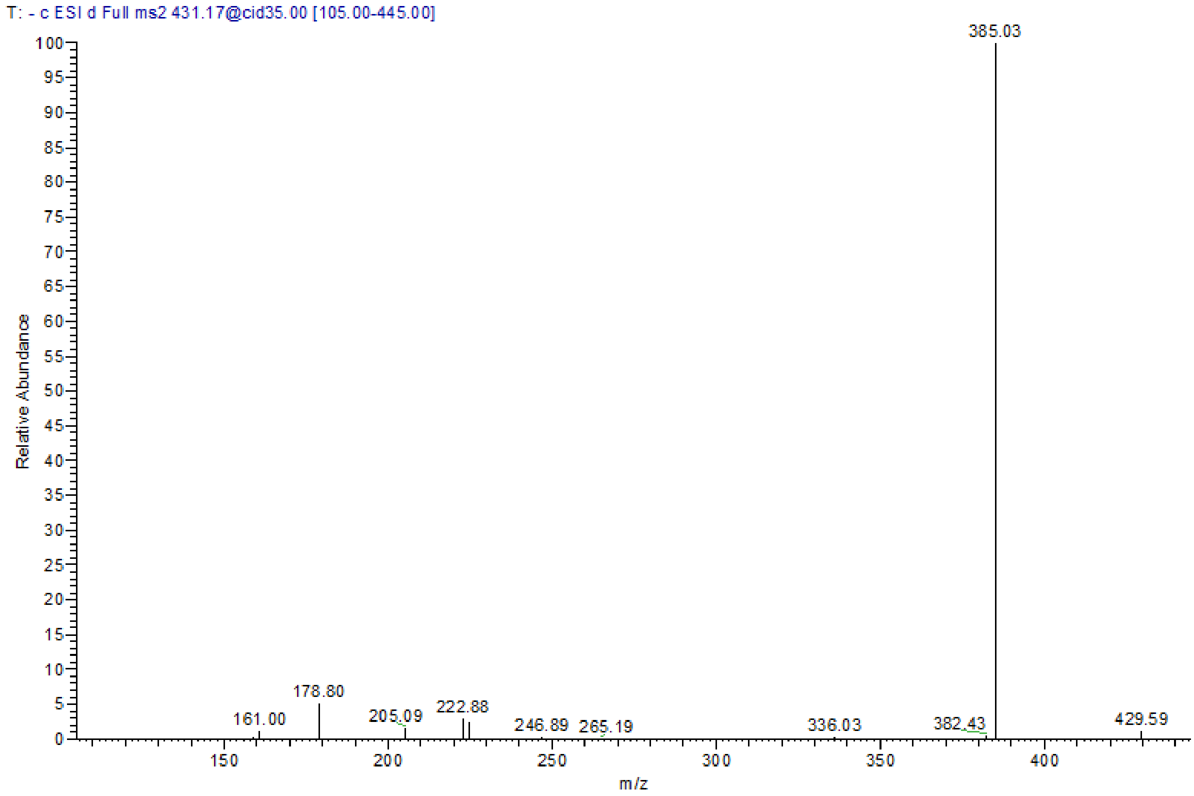

Figure S10. MS/MS profile of sinapic acid 3-*O*- hexoside [M-H + 46, formic acid adduct]

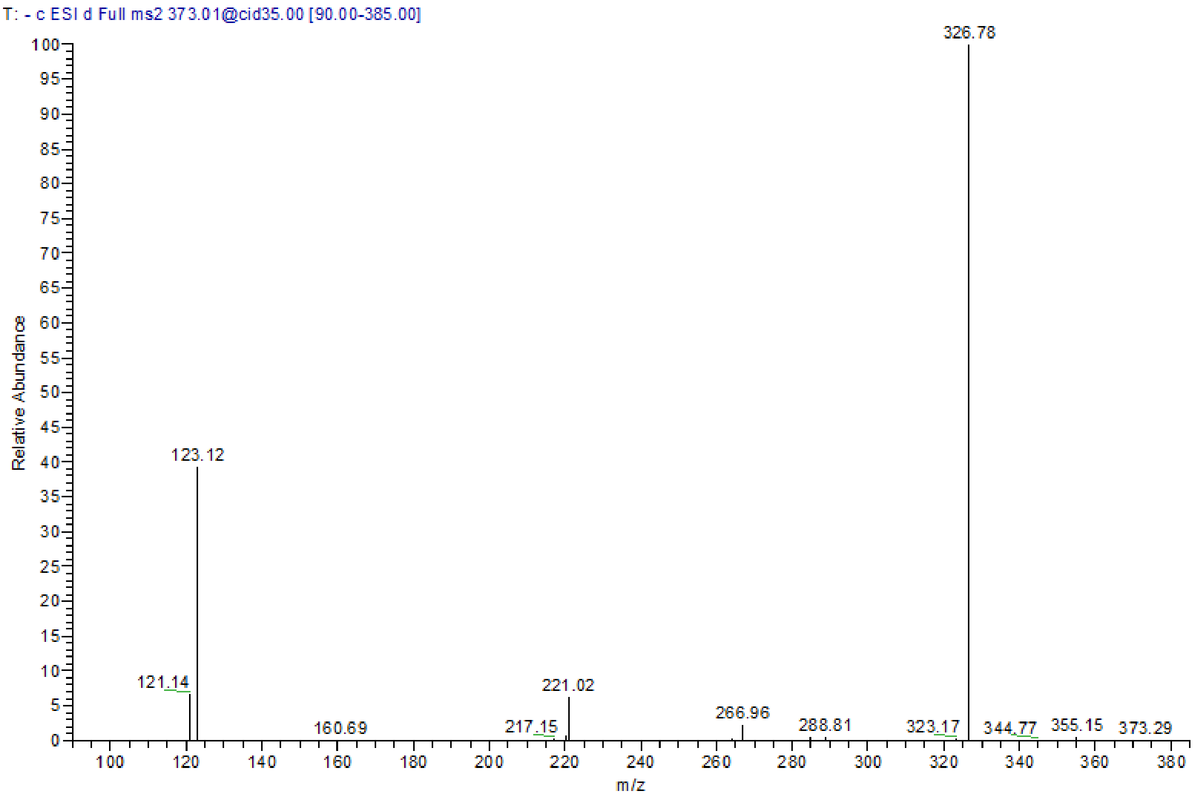

Figure S11. MS/MS profile of 2’-*O*-acetyl-salicin [M-H + 46, formic acid adduct]

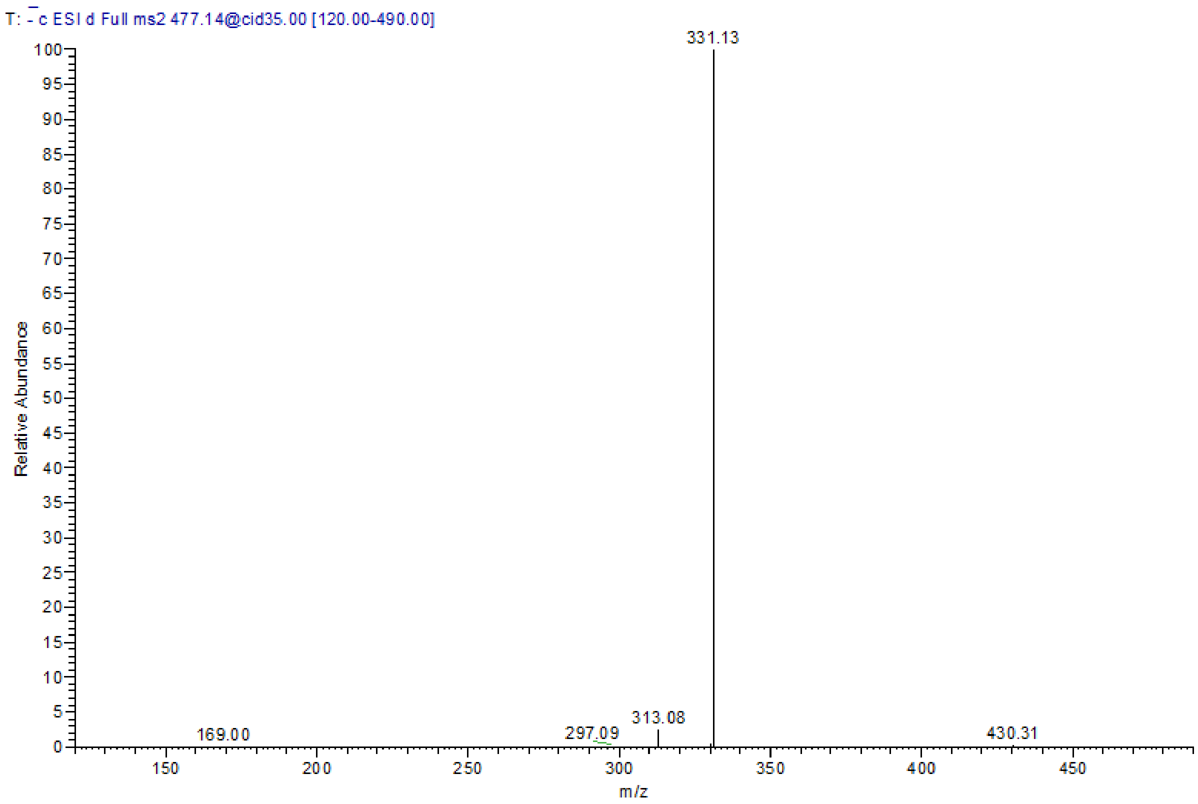

Figure S12. MS/MS profile of coumaroylgalloyl glucose

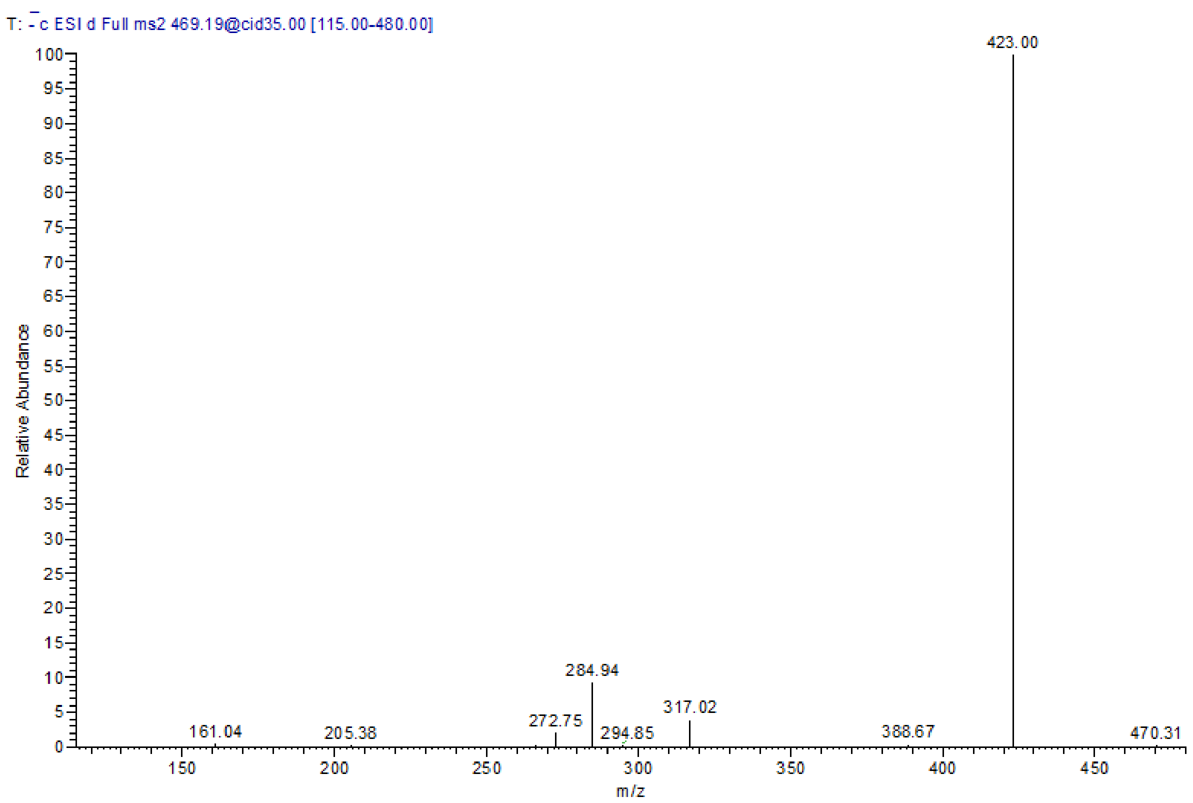

Figure S13. MS/MS profile of salicortin [M-H + 46, formic acid adduct]

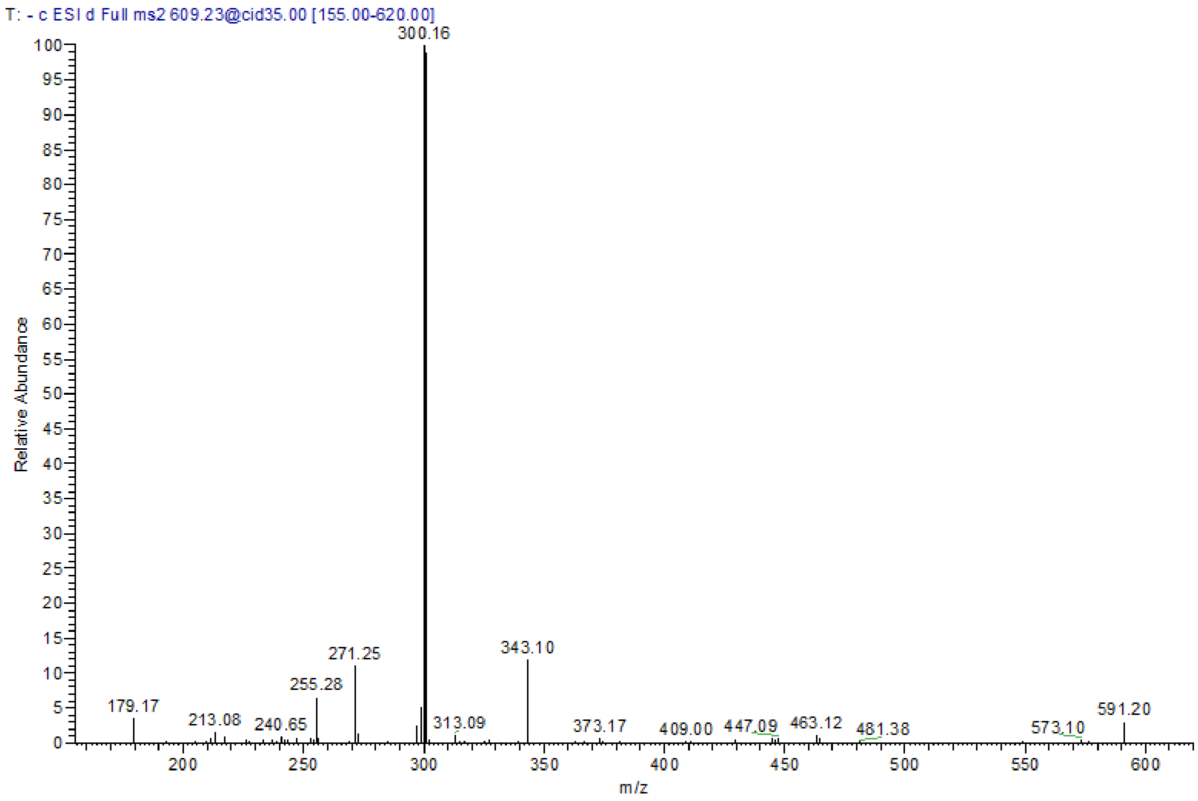

Figure S14. MS/MS profile of rutin

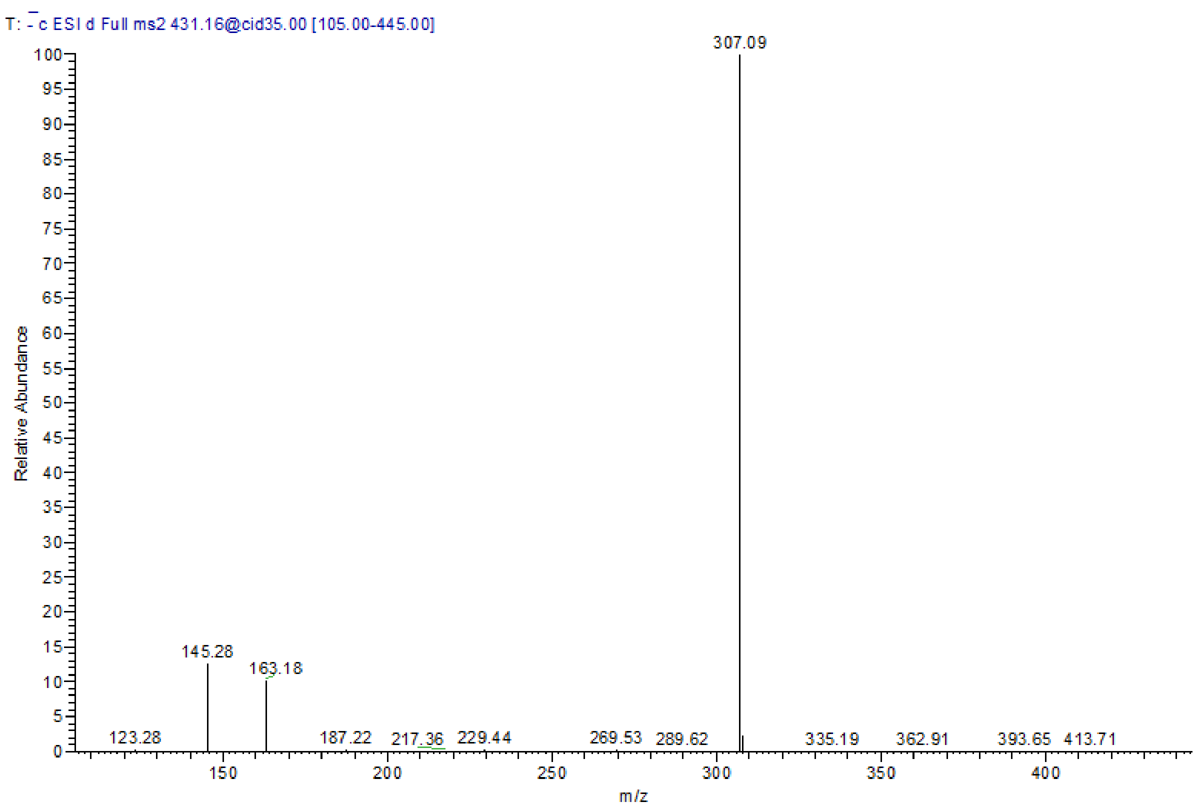

Figure S15. MS/MS profile of trichocarposide

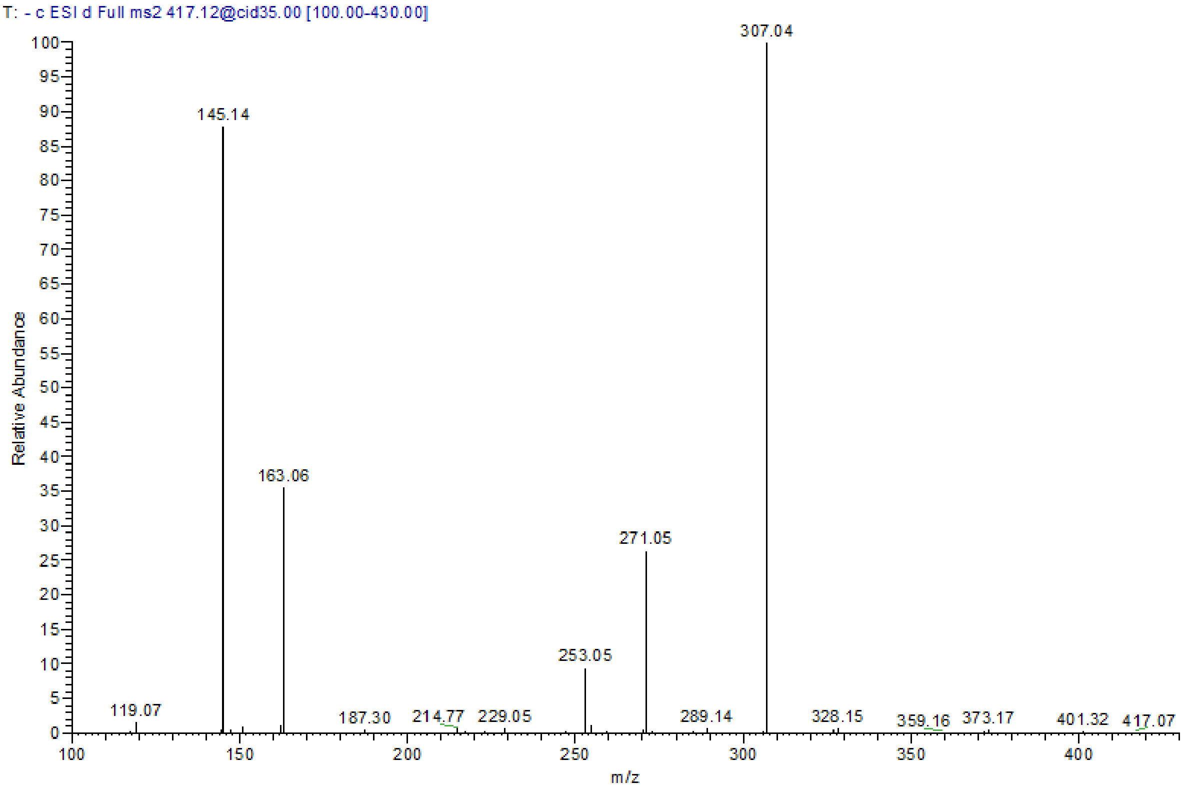

Figure S16. MS/MS profile of dihydrocinnamoyl salicin

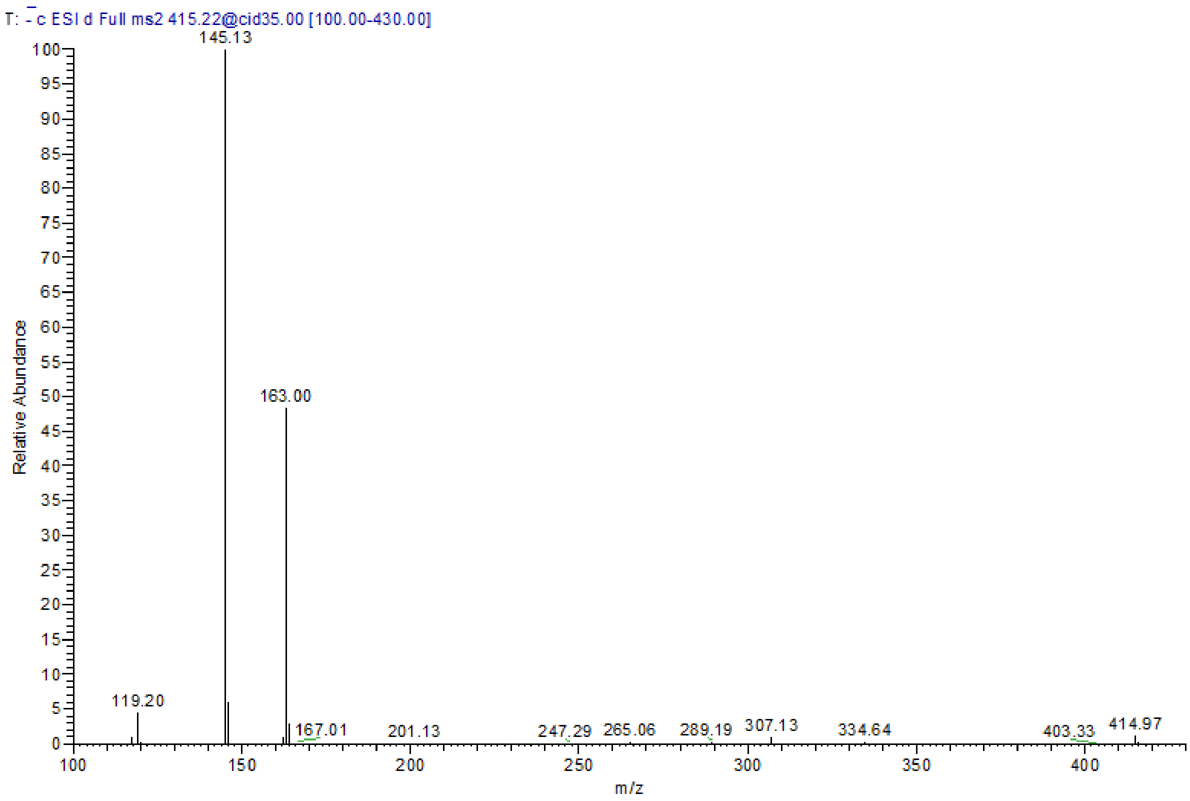

Figure S17. MS/MS profile of cinnamoyl salicin

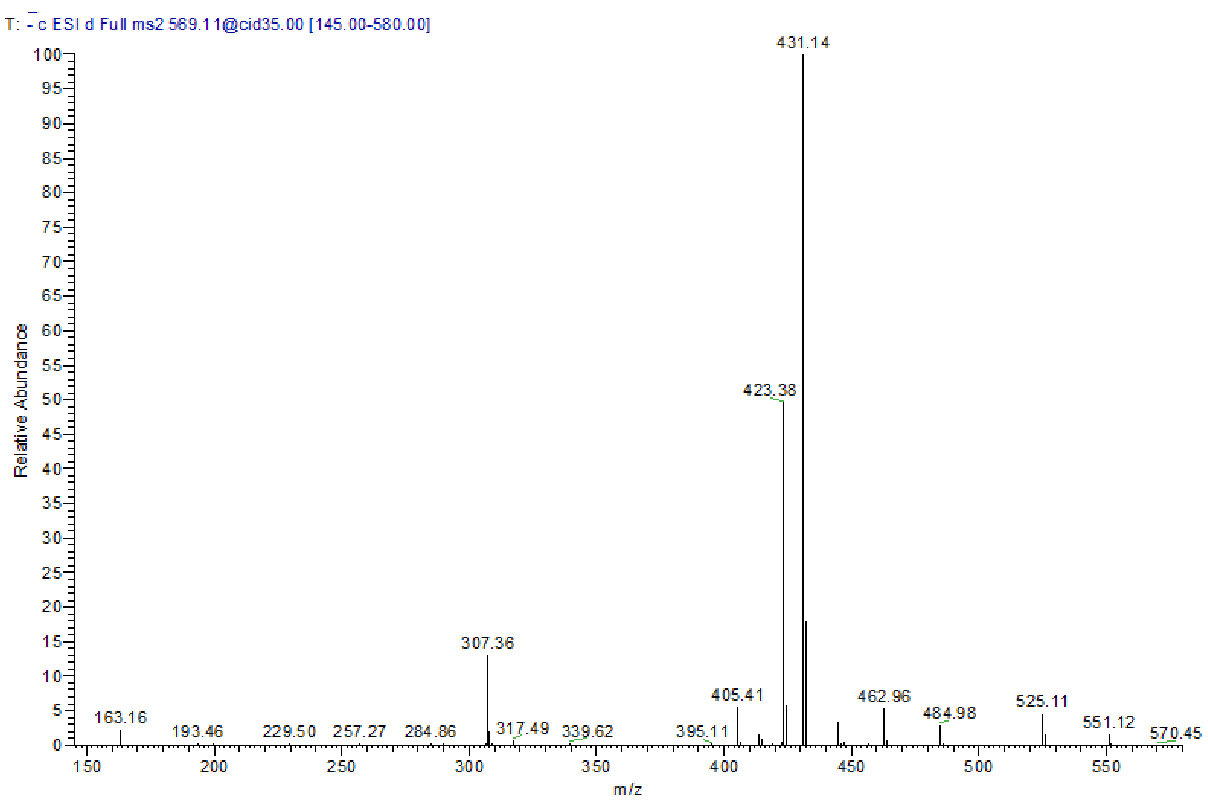

Figure S18. MS/MS profile of coumaroyl dihydrobenzoyl salicin

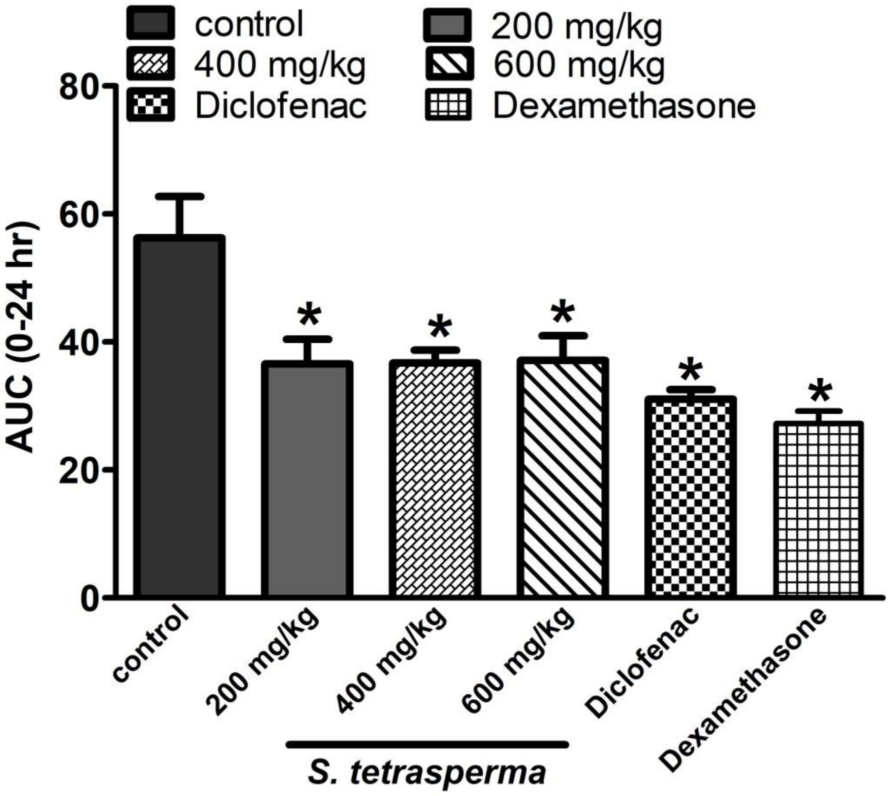

Figure S19. Effect of 1 h prior administration of *S. tetrasperma* (200, 400 and 600 mg/kg, p.o.), diclofenac sodium (20 mg/kg, p.o) or dexamethasone (2 mg/kg, p.o) on carrageenan (1% suspension, 0.1 ml/rat) induced-hind paw edema in rats. Edema thickness (mm) was measured before and then hourly for 5 h and at 24 h after carrageenan injection. Data represents the AUC_{0-24} and is expressed as mean ± S.E.M (n=5). *$p < 0.05$ vs. control values.

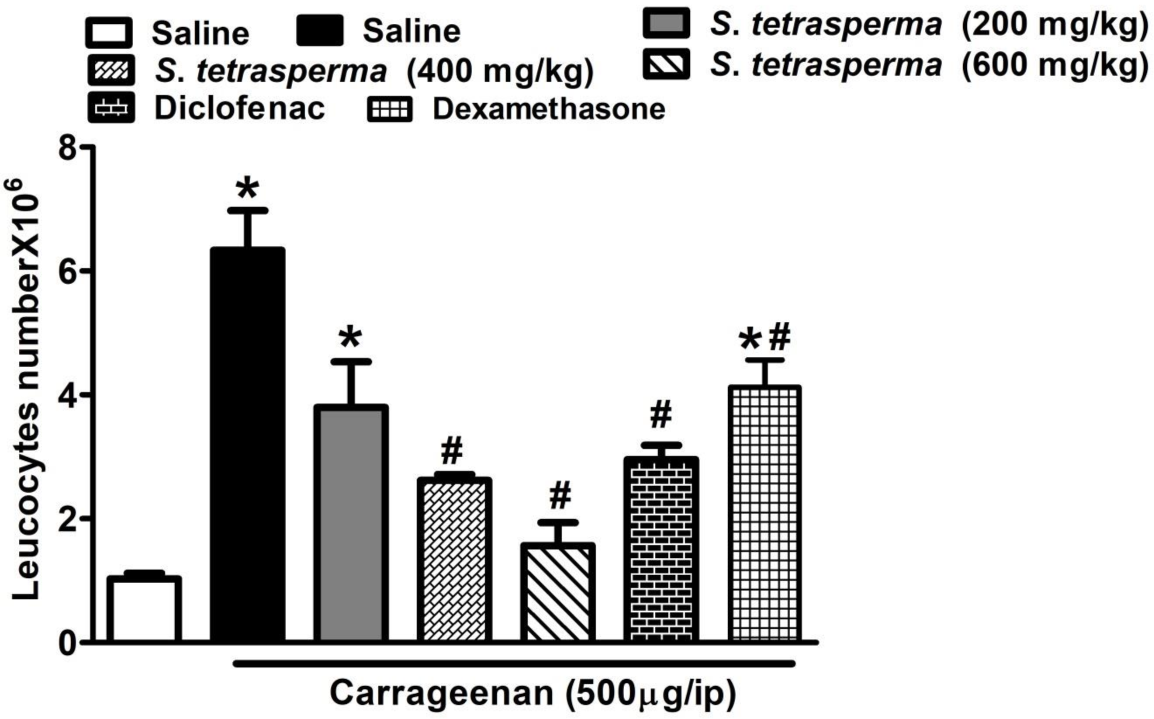

Figure S20. Effect of carrageenan (500 µg, ip) on leukocytes migration into the peritoneal cavity in mice (total number X10^6) with or without 1h prior treatment with *S. tetrasperma* (200, 400 and 600 mg/kg, p.o.), diclofenac sodium (20 mg/kg, p.o) or dexamethasone (2 mg/kg, p.o). Data is expressed as mean ± S.E.M (n=5-7). *$p < 0.01$ vs. vehicle (saline) values, $^{\#}p < 0.01$ vs control (carrageenan treated group).

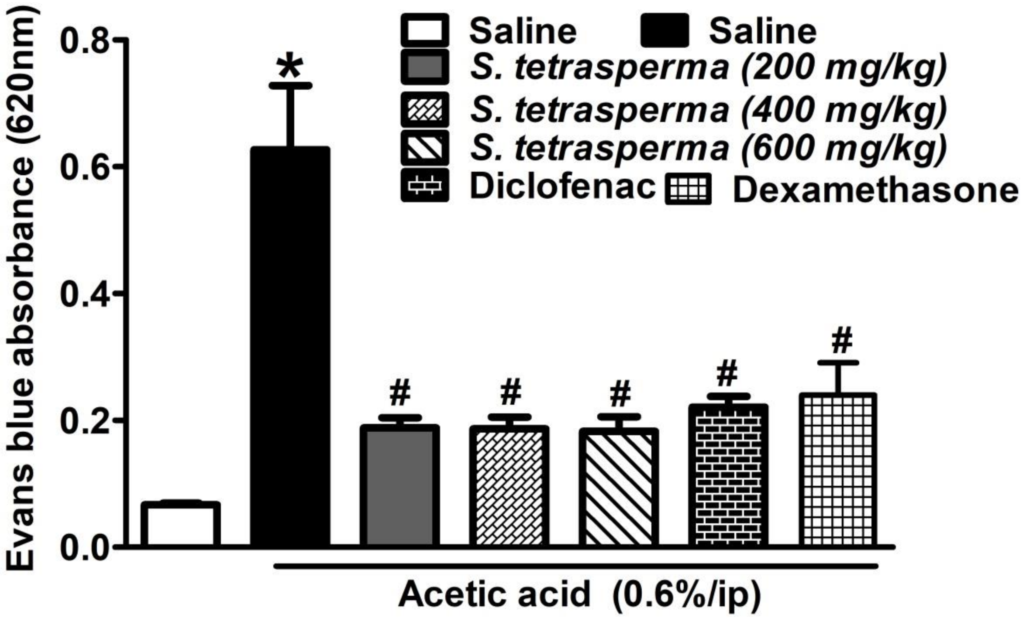

Figure S21. Effect of *S. tetrasperma* extract (200, 400 and 600 mg/kg, p.o.) on acetic acid-induced vascular permeability. The amount of Evans blue dye in the abdominal cavity was measured as an indicator of inflammation degree. The values are expressed as the means ± SEM (n=5-6). * p<0.001 compared to saline group. $^{\#}p$ <0.001 compared to control (acetic acid only treated group).

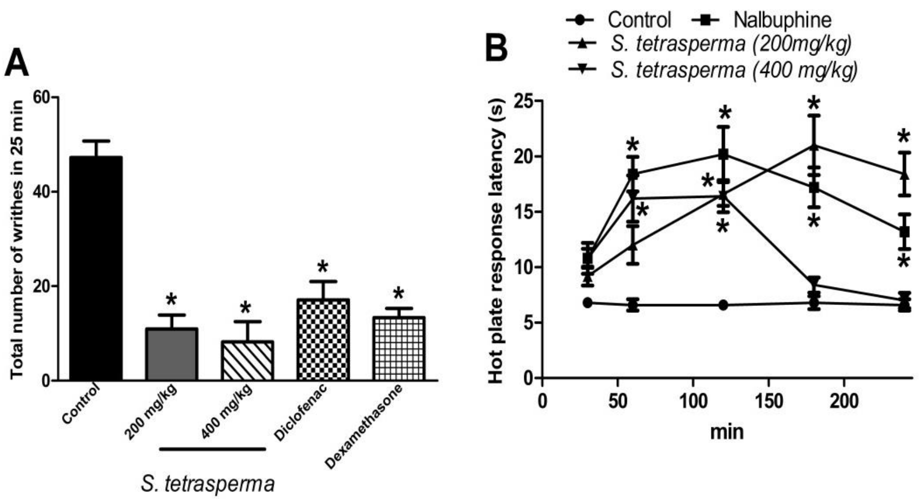

Figure S22. Effect of *S. tetrasperma* (200 and 400 mg/kg, p.o.), diclofenac (20 mg/kg, p.o) or dexamethasone (2 mg/kg, p.o) on acetic acid-induced writhing (0.7%, 1 ml/100 g) in mice (**A**). Hot plate response latency (s) measured 1–4 h after vehicle, the extract (200 and 400 mg/kg, p.o.) or nabuphine (10mg/kg, p.o) administration in mice (**B**). Data is expressed as mean ± S.E.M (n=5-8). *$p < 0.001$ vs. control values.

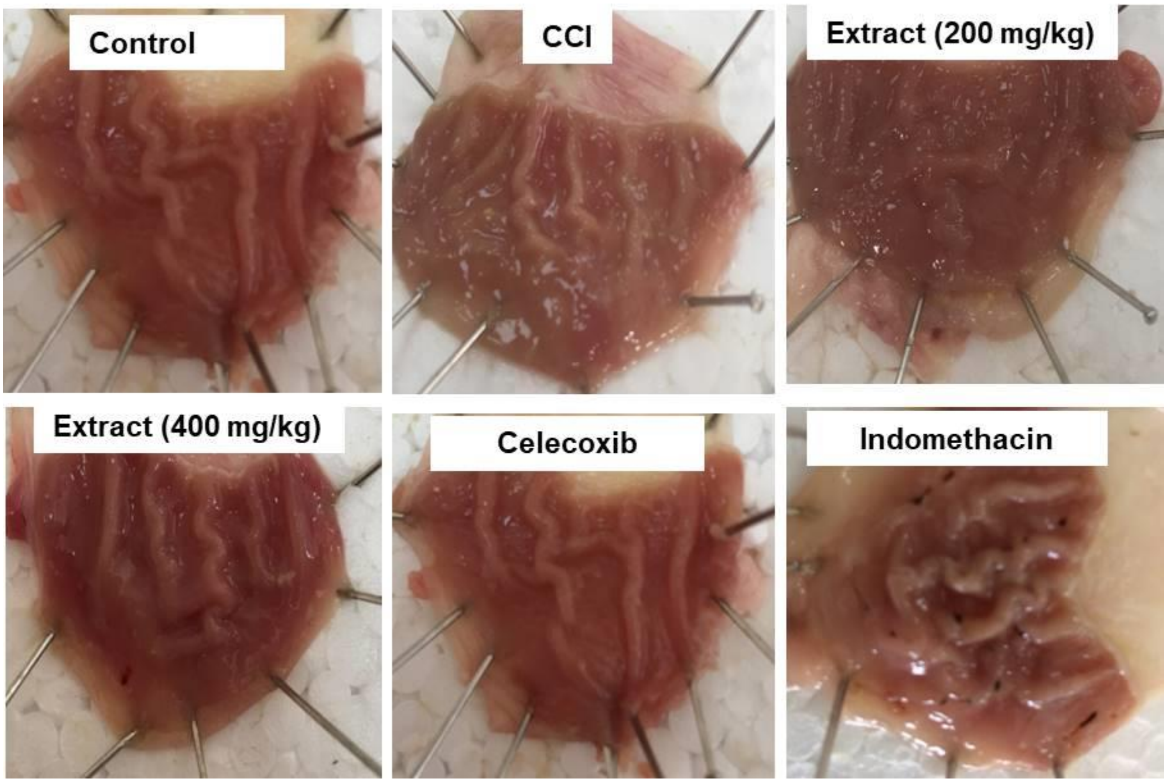

Figure S23. Representative stomach images obtained from CCI rats receiving either the vehicle or different treatments

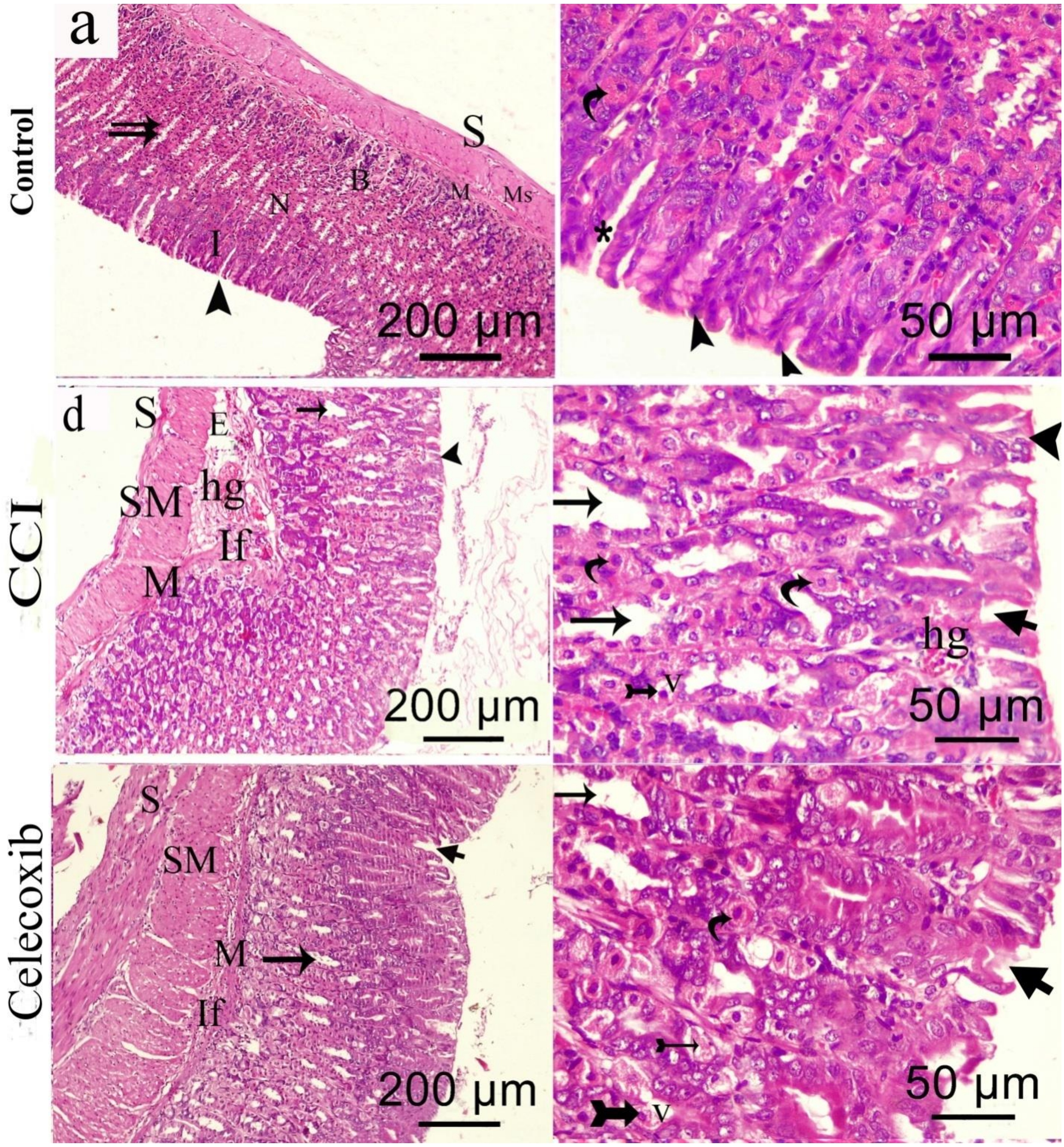

Control
a
S
B
N
M
Ms
I
200 µm
*
50 µm
CCI
d
S
E
SM
hg
If
M
200 µm
hg
v
50 µm
Celecoxib
S
SM
M
If
200 µm
v
50 µm

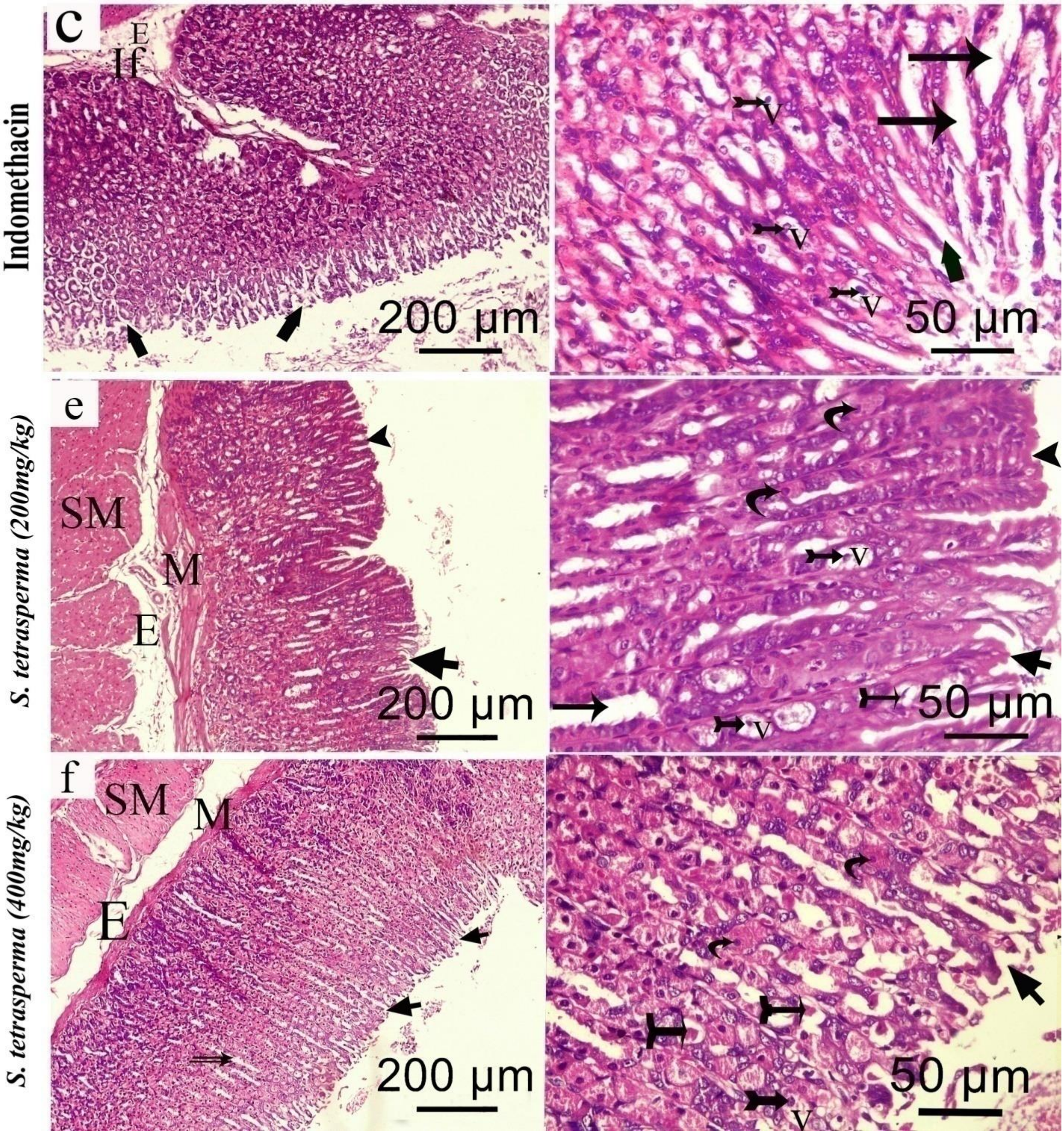

Figure S24. Representative photomicrograph of stomach sections (fundic mucosa) from different groups, (a) control; (b) CCI; (c) celecoxib; (d) indomethacin; (e) *S. tetrasperma* (200 mg/kg) and (f) *S. tetrasperma* (400 mg/kg). The arrow heads, double arrow, arrow, curved arrows, thin bifid arrow, thick bifid arrows and twisted arrows, short arrow, thick arrow and astrexic illustrate normal fundic mucosa, normal fundic glands, wide fundic glands, normal parietal cell, remnant of cytoplasm of parital cells, dark stained nuclei of parital cell, glandular

cells with karyolitic nuclei, slight shedingfundic mucosa, erosion of fundic mucosa and gastric pit respectively. Isthmus (I), N (neck), basic region (B), mucosa (M), muscularis mucosa (Ms), serosa (S), edema (E), infilteration (If), hemorrhage (hg), vacuoles (v) and blood vessel (bv) Scale bar, 200 μm ×100, 50 μm x400.

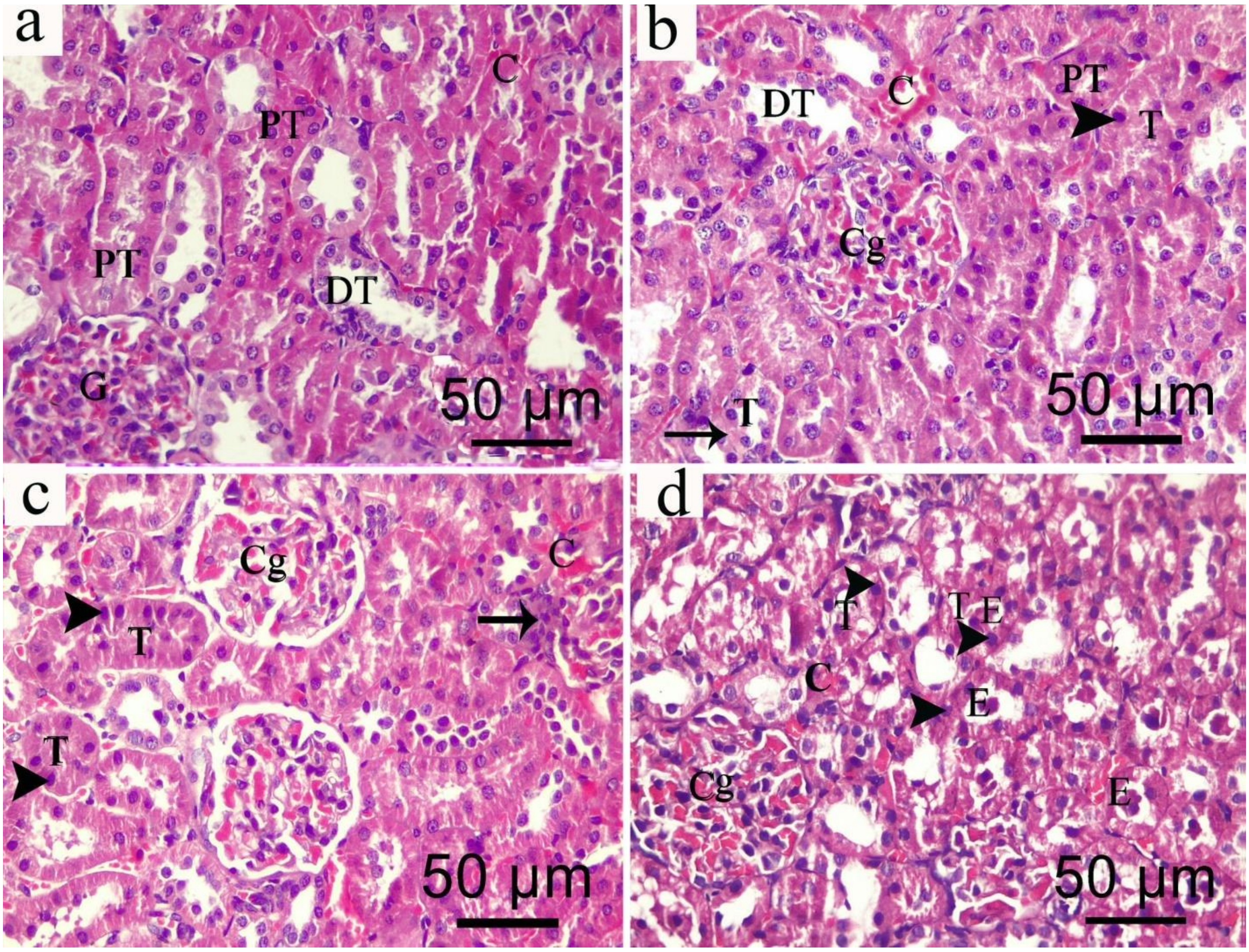

Figure S25. Representative photograph of renal cortex sections of the different groups. (a) control; (b) CCI; (c) *S. tetrasperma* (200 mg/kg); (d) *S. tetrasperma* (400 mg/kg). Arrow and arrow head illustrate the dark stained nuclei and infilterating cells respectively. G, glomerulus; Cg, congested glomeruli; PT, proximal convoluted tubule; DT, distal convoluted tubule; T, affected tubule; E, exfoliated cells; C, peritubular capillaries.

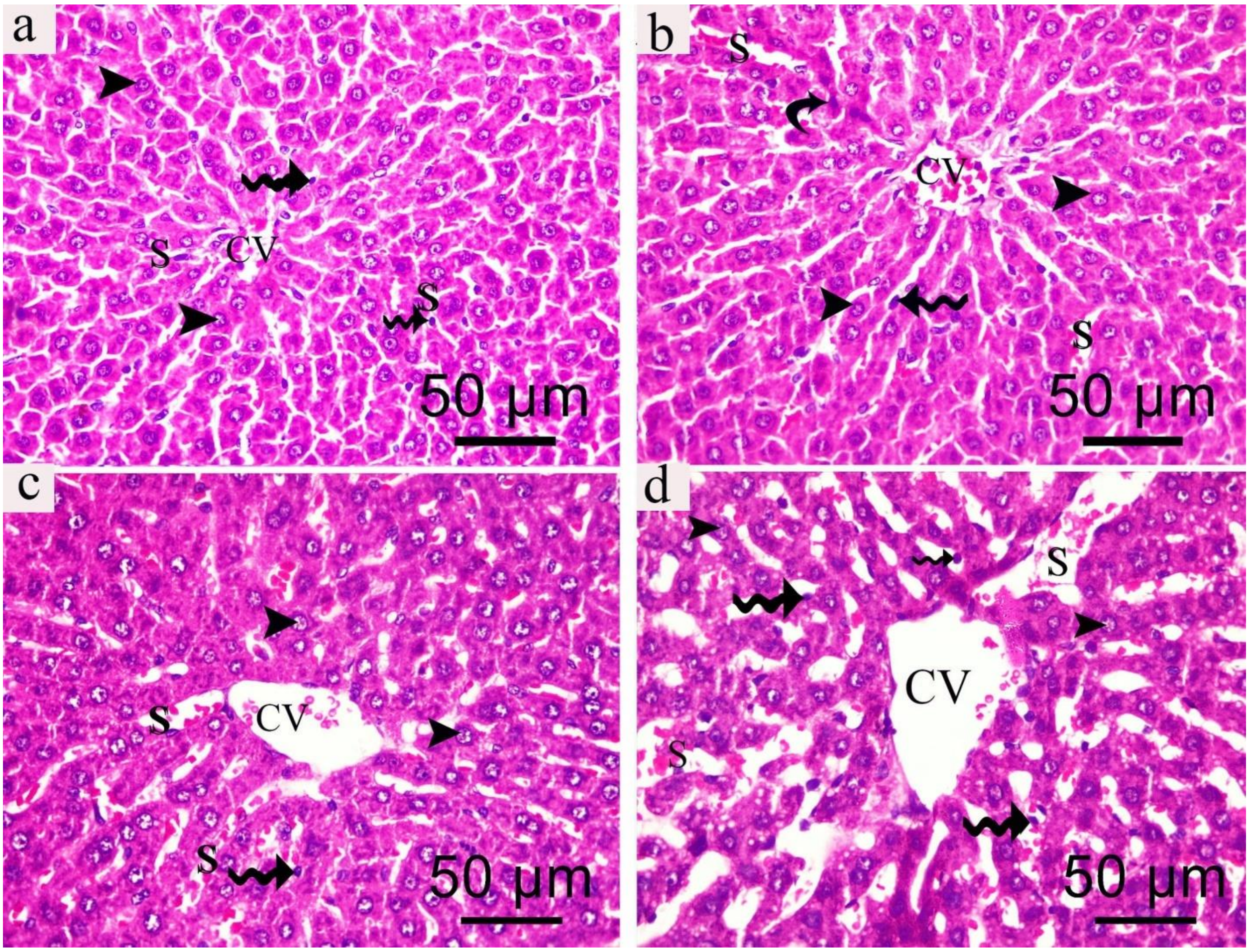

Figure S26. Reprehensive photograph in sections of liver from different groups, (a) control; (b) CCI; (c) *S. tetrasperma* (200 mg/kg); (d) *S. tetrasperma* (400 mg/kg). Arrow head, curved arrow and twisted arrow illustrate normal hepatocytes, Kupffer cells, hepatocytes with dark stained nuclei infiltrations respectively. S, the blood sinusoids; Cv, the central vein, Scale bar, 50 µm ×400.

References

Abdelall, E. K. A., P. F. Lamie, et al. (2016). "Cyclooxygenase-2 and 15-lipoxygenase inhibition, synthesis, anti-inflammatory activity and ulcer liability of new celecoxib analogues: Determination of region-specific pyrazole ring formation by NOESY." Bioorg Med Chem Lett26(12): 2893-2899.

Lee, D. Y., B. K. Choo, et al. (2009). "Anti-inflammatory effects of Asparagus cochinchinensis extract in acute and chronic cutaneous inflammation." J Ethnopharmacol121(1): 28-34.

Liu, C., H. Su, et al. (2017). "Forsythoside A exerts antipyretic effect on yeast-induced pyrexia mice via inhibiting transient receptor potential vanilloid 1 function." Int J Biol Sci13(1): 65-75.

Sengar, N., A. Joshi, et al. (2015). "Anti-inflammatory, analgesic and anti-pyretic activities of standardized root extract of Jasminum sambac." J Ethnopharmacol160: 140-148.

Sobeh, M., M. F. Mahmoud, et al. (2018). "Syzygium aqueum: a polyphenol- rich leaf extract exhibits antioxidant, hepatoprotective, pain-killing and anti-inflammatory activities in animal models." Frontiers in Pharmacology9(566).

Kim, C. S., Subedi, L., Park, K. J., Kim, S. Y., Choi, S. U., Kim, K. H., Lee, K. R., 2015. Salicin derivatives from *Salix glandulosa* and their biological activities. Fitoterapia, 106, 147–152. doi:10.1016/j.fitote.2015.08.013

Boeckler, G. A., Gershenzon, J., Unsicker, S. B., 2011. Phenolic glycosides of the Salicaceae and their role as anti-herbivore defenses. Phytochemistry 72, 1497–1509

Mizuno, M., Kato, M., Misu, C., Iinuma, M., Tanaka, T., 1991. Chaenomeloidin: A phenolic glucoside from leaves of *Salix chaenomeloides*. J. Nat. Prod. 54, 1447-1450

Tanagornmeatar, K., Chaotham, C., Sritularak, B., Likhitwitayawuid, K., Chanvorachote, P., 2014. Cytotoxic and anti-metastatic activities of phenolic compounds from *Dendrobium ellipsophyllum*. Anticancer Res. 34, 6573-6579.

Nybakken, L., Julkunen-Tiitto, R., 2013. Gender differences in *Salix myrsinifolia* at the pre-reproductive stage are little affected by simulated climatic change. Physiol. plant. 147, 465-476.

Kumari, A., Upadhyay, N. K., Khosla, P. K., 2016. Gender specific variation of two phenolic glycosides (populin and salicin) in *Populus ciliata* and identification of a new compound (cinnamoyl-salicin). Int. J. Pharm. Pharm. Sci. 8, 156-162 DOI: 10.22159/ijpps.2016v8i12.14843

Keefover-Ring, K., Ahnlund, M., Abreu, I. N., Jansson, S., Moritz, T., Albrectsen, B. R., 2014. No evidence of geographical structure of salicinoid chemotypes within *Populus tremula*. PLoS One *9*, e107189.

Gligorić, E., Igić, R., Suvajdžić, L., Grujić-Letić, N., 2019. Species of the genus Salix L.: Biochemical screening and molecular docking approach to potential acetylcholinesterase inhibitors. Appl. Sci. 9, 1842. doi:10.3390/app9091842

Bakour, M., Fernandes, Â., Barros, L., Sokovic, M., Ferreira, I. C., 2019. Bee bread as a functional product: Chemical composition and bioactive properties. LWT 109, 276-282.

Xu, C. L., Zheng, Y. N., Yang, X. W., Li, X. G., Li, X., Chen, Q. C., 2007. Raddeanalin, a new flavonoid glycoside from the leaves of *Salix raddeana* Laksh. *J. Asian Nat. Prod. Res. 9*, 415-419.

Löffler, C., Czygan, F. C., Proksch, P., 1997. Phenolic constituents as taxonomic markers in the genus *Cuscuta* (Cuscutaceae). *Biocchem. Syst. Ecol. 25*, 297-303.

Przybyłek, I., Karpiński, T. M., 2019. Antibacterial properties of propolis. Molecules, 24, 2047.

Thapliyal, R. P., Bahuguna, R. P., 1993. Fatty acids and flavonoids of *Salix lindleyana*. *Int. J. Pharmacogn. 31*, 165-166.

Ahmad, V. U., Burki, A. M., Mahmoud I., Smith, D.L., 1984. Chemical constituents of *Blepharis sindica* seeds. J. Chem. Soc. Pakistan 6, 217-223.

Chechetkin, I. R., Blufard, A. S., Yarin, A. Y., Fedina, E. O., Khairutdinov, B. I., Grechkin, A. N., 2019. Detection and identification of complex oxylipins in meadow buttercup (*Ranunculus acris*) leaves. Phytochemistry *157*, 92-102.

El-Wakil, E.A., Abdel-Hameed, E. S., El-Sayed, M.M., Abdel-Lateef, E.E., 2015. Identification of the chemical composition of the methanolic extract of *Salix tetrasperma* Roxb. using LC-ESI-MS and evaluation its potential as antioxidant agent. Der Pharma Chem. 7, 168-177.

Pobłocka-Olech, L., Krauze-Baranowska, M., Głód, D., Kawiak, A., Łojkowska, E., 2010. Chromatographic analysis of simple phenols in some species from the genus Salix. Phytochem. Anal. 21, 463-469.

Csernatoni, F. Baciu, A.; Pop, R.;Romanciuc, F.; Socaciu, C. 2014. Characterization of seven medicinal plants included in an original formula PROMEN, to prevent prostate diseases. Hop Med. Plants 22, 70-78.

Nićiforović, N., Abramovič, H., 2014. Sinapic acid and its derivatives: natural sources and bioactivity. Compr. Rev. Food Sci. Food Saf. 13, 34-51.

Wang, D. M., Pu, W. J., Wang, Y. H., Zhang, Y. J., Wang, S. S., 2012. A new isorhamnetin glycoside and other phenolic compounds from *Callianthemum taipaicum*. *Molecules*, *17*(4), 4595-4603.

Barros, L., Dueñas, M., Pinela, J., Carvalho, A. M., Buelga, C. S., Ferreira, I. C., 2012. Characterization and quantification of phenolic compounds in four tomato (*Lycopersicon esculentum* L.) farmers' varieties in northeastern Portugal homegardens. Plant Food Hum. Nut. 67, 229-234.

Sobral, F., Calhelha, R., Barros, L., Dueñas, M., Tomás, A., Santos-Buelga, C., Ferreira, I., 2017. Flavonoid composition and antitumor activity of bee bread collected in northeast Portugal. *Molecules*, *22*, 248.
